# 3D Bioprinted Chitosan-Based Hydrogel Scaffolds in Tissue Engineering and Localised Drug Delivery

**DOI:** 10.3390/pharmaceutics14091978

**Published:** 2022-09-19

**Authors:** Maria Lazaridou, Dimitrios N. Bikiaris, Dimitrios A. Lamprou

**Affiliations:** 1 Laboratory of Chemistry and Technology of Polymers and Dyes, Department of Chemistry, Aristotle University of Thessaloniki, 54124 Thessaloniki, Greece; 2 School of Pharmacy, Queen’s University of Belfast, 97 Lisburn Road, Belfast BT9 7BL, UK

**Keywords:** 3D bioprinting, bioink, biomaterials, chitosan, tissue engineering, drug delivery

## Abstract

Bioprinting is an emerging technology with various applications in developing functional tissue constructs for the replacement of harmed or damaged tissues and simultaneously controlled drug delivery systems (DDSs) for the administration of several active substances, such as growth factors, proteins, and drug molecules. It is a novel approach that provides high reproducibility and precise control over the fabricated constructs in an automated way. An ideal bioink should possess proper mechanical, rheological, and biological properties essential to ensure proper function. Chitosan is a promising natural-derived polysaccharide to be used as ink because of its attractive properties, such as biodegradability, biocompatibility, low cost, and non-immunogenicity. This review focuses on 3D bioprinting technology for the preparation of chitosan-based hydrogel scaffolds for the regeneration of tissues delivering either cells or active substances to promote restoration.

## 1. Introduction

**Figure 1:**
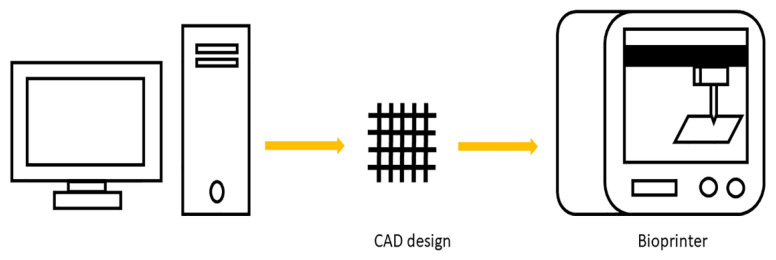
Three-dimensional printing process.

Additive manufacturing (AM), widely known as 3D printing (3DP), is attributed to the layer-by-layer addition or deposition of a material or different materials in order to build a three-dimensional (3D) construct. Three-dimensional printing, also known as rapid prototyping (RP), uses computer data such as computer-assisted design (CAD), which can be produced using computer tomography (CT), or magnetic resonance imaging (MRI), and translates them into constructed 3D objects (Figure 1). Because of its capacity for structures with complex features in the interior part, 3DP technology has been used in several fields, such as engineering, manufacturing, aerospace, automotive, jewelry, arts, and architecture, among many other fields.

**Figure 2:**
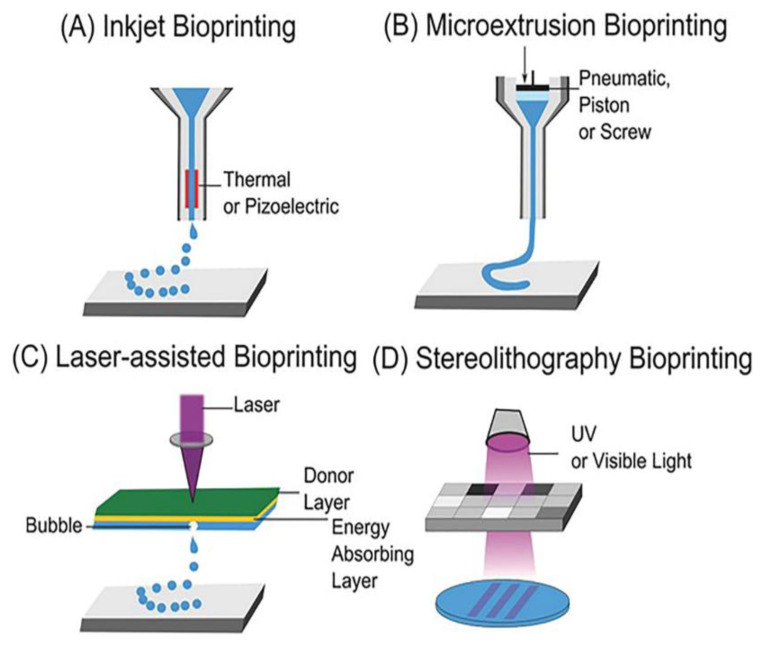
Representation of the main 3D Bioprinting technologies. (**A**) Inkjet Bioprinting, (**B**) Micro extrusion Bioprinting, (**C**) Laser-assisted Bioprinting, and (**D**) Stereolithography Bioprinting. Reproduced from [2], Copyright 2018, Wiley.

In the early 2000s, there was the appearance of fabrication of scaffolds including cells within the structure of their 3DP matrices, which would enable different cells to be printed at certain sites, with the arrangement needing increased significance, and this process was called bioprinting. According to predictions, by 2024, the market of the 3D printing industry will have reached the size of USD 35 billion [1]. A number of different 3DP techniques have been developed until now (Figure 2), such as inkjet bioprinting, extrusion-based bioprinting, laser-assisting bioprinting, and stereolithography (SLA)-based bioprinting [2].

The reliable definition of the term bioink became an issue of debate between members of the scientific community. The term ‘bioink’ was first used to refer to organ printing in 2003 and was introduced together with the term hydrogel membrane. In the beginning, the concept was to afford or even print a biopaper (hydrogel) and then introduce viable cells or tissue spheroids [3]. Cells and cell aggregates were used as the bioink [4,5]. In order to make clear the distinction, (bio-) materials that can be printed and subsequently seeded with cells after printing, but which are not directly developed with cells, do not qualify as bioink. It is suggested that these should be defined as biomaterial inks [3]. Such biomaterial inks may be used as scaffolds for cell seeding, bioreactors, or implants, or they may be used at the same time for bioink fabrication in hybrid approaches for mechanical maintenance [6,7]. On the other hand, some of the existing articles suggest an extension of the definition of additively manufactured materials.

**Figure 3:**
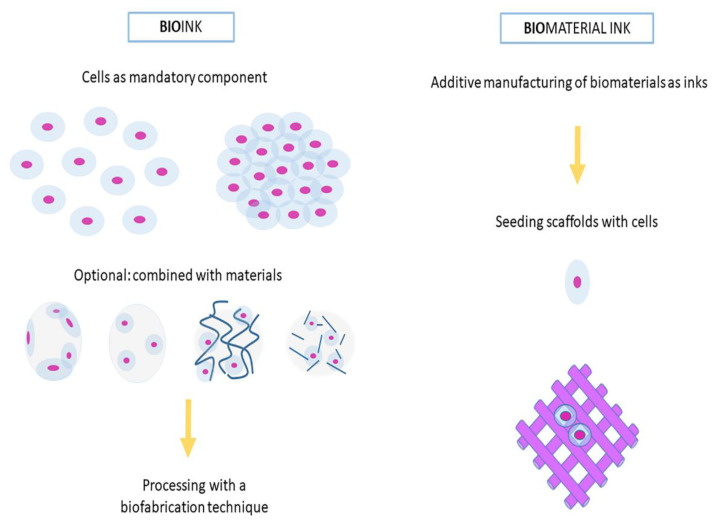
Distinction between a bioink (**left**), where cells are a mandatory component of the printing formulation as single cells, coated cells and cell aggregates (of one or several cell types), or in combination with materials (for example seeded or formulated in a physical hydrogel) and a biomaterial ink (**right**), where a biomaterial is used for printing and cell-contact takes place post-fabrication.

For bioprinting technology, the key requirement is developing a suitable ink material. Based on the cell insertion methodology, ink material can be classified as bioink and biomaterial-ink (Figure 3). In bioink, cells are loaded in the ink material, and cell printing is carried out. However, in biomaterial-ink, hydrogels are printed, and post-printing cells are seeded externally on the printed materials. This method provides flexibility in selecting raw materials and eliminates the printing-process-induced impact on the viability of cells. Paxton et al. highlighted that inks intended to be used for extrusion-based bioprinting must congregate the rheological demands. Apart from biocompatibility and shear thinning abilities, hydrogels also require a fast crosslinking strategy to maintain their structural fidelity [8].

**Figure 4:**
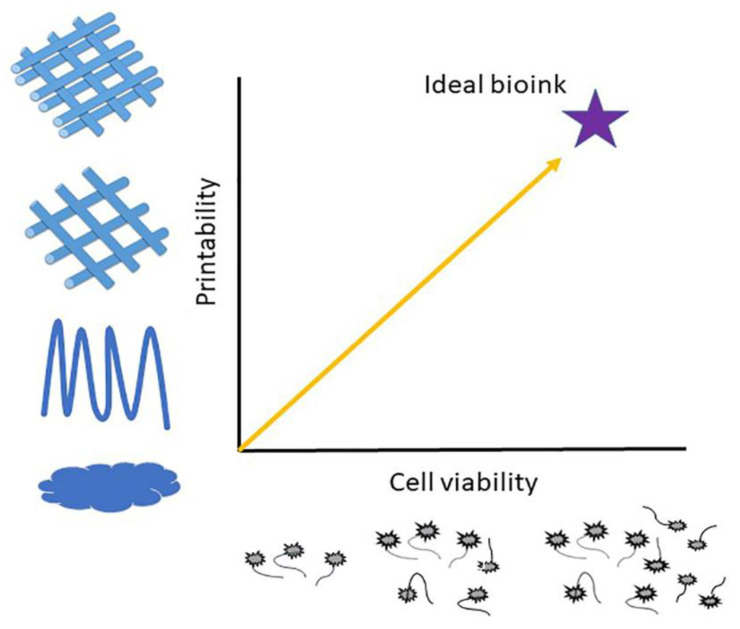
The biofabrication window for biomaterials.

An ideal bioink, in order to be suitable for the printing process, should have some fundamental properties. The most essential properties of hydrogels used as bioinks are biocompatibility, printability, mechanical property, hydrophilicity, geometric structure, and biodegradability. Additionally, bioinks are required to cause a sol-gel behavior, eliminating the processing time, and many chemical and physical crosslinking mechanisms are used to achieve high accuracy of the structure and stability. The ‘biofabrication window’ present this compromise between suitability for fabrication and capacity of cell accommodation (Figure 4).

Natural polymers such as chitosan, especially in its hydrogel form, play a key role as bioink for 3D bioprinting of tissues and drug delivery systems. Chitosan as biopolymer material is abundant in nature and is acquired from chitin, the second most abundant natural polymer after cellulose, which is an N-acetyl glucosamine polymer existing on exoskeletons and shells of crustaceans, especially from crabs and shrimps. Chitin deacetylation takes place via acidic and basic treatments of chitin. Chitosan is a material that has more than 50% acetyl groups of chitin, removed and replaced by amine groups. Although chitin is not soluble in aqueous media, chitosan is soluble in acidic solutions. Among others, its non-toxicity and its Food and Drug Administration (FDA) approval for pharmaceutical applications have attracted interest [9,10]. The presence of the amino group along D-glucosamine residues, protonated in acidic media, can clarify most of the chitosan properties. Mucoadhesion, for example, can be explained by the interaction between the protonated amino groups and the negatively charged moieties in the mucin, coming from sialic acid, the main protein that constitutes the mucus. Furthermore, the hemostatic activity of chitosan comes from the interaction of protonated amino group with the negative charge existing on the membrane of red blood cells, unlike chitin. The positive charges of chitosan can also lead to a restructuring of the proteins on the cell membrane and thus enhance permeation ability. Except for that, this could avoid the entrance of fundamental nutrients for cell survival to enter the cell resulting in antimicrobial activity of chitosan. Another mechanism responsible for the antimicrobial activity of chitosan could be the capability of binding with the DNA of the cell so as to prevent RNA synthesis and cell proliferation. Concerning biodegradability, chitosan also affords breakable glycosidic bonds by several proteases, such as lysozyme, which can come to non-toxic residues and subsequently be excreted. In the meantime, eight chitinases have been identified in the human body. However, the degradation rate is mainly related to the DD, where crystallinity is maximum for 100% deacetylated chitosan. It is indicated that the increase in crystallinity is combined with the decrease in degradation rate [11].

Many efforts have been reported in the literature for the fabrication of chitosan-based scaffolds by utilizing traditional methods. However, low mechanical properties of the electrospinning method, solvent residuals in the final formulation and limited control on the pore size in case of solvent casting, and finally, energy and time consumption with the presence of harmful solvents with irregular pore structures in freeze drying have led to the need for the investigation of alternatives. On the contrary, the 3D printing technique helped to overcome this limitation, improving the spatial control of micro-architecture and spatial content. Moreover, 3D bioprinting can be used to deposit living cells, extracellular matrices, and other biomaterials with user-defined fined patterns to build complex constructs ‘from the bottom up’. The perspective of creating vascular structures is also enhanced by bioprinting as internal channels with vascular cells can be printed into constructs promoting the development of blood vessels in vivo.

Most applications and research studies on chitosan focus on its hydrogel form [12]. Hydrogels are commonly used as a bioink material in scaffold-based bioprinting as a result of their numerous attractive characteristics that make the 3DP process more fulfilled. The main reasons are their biodegradability, biocompatibility, and the existence of cell-binding sites for cell attachment, proliferation, or differentiation. In general, chitosan is soluble in aqueous acid media at pH 5–6 at 24 °C, and gelation can occur through several techniques such as ionotropic, cross-linker-assisted, polyelectrolyte complexed, or self-assembled.

Chitosan is often combined with other natural (e.g., cellulose, gelatin, silk, alginate, hyaluronic acid, and collagen) or synthetic (e.g., PCL, poly(ethylene glycol)) polymers to add extra properties. Moreover, chitosan has functional groups that can be modified to enhance its properties, notably its low mechanical properties, or utilized for the synthesis of polymer/active substance conjugates [13].

In this review, a brief discussion of different chitosan-based formulations currently employed for printing and bioprinting will be provided. The discussion will not be limited to the 3DP cell-laden formulations but also to the 3DP formulations where the cells are inserted after printing. Additionally, the review concludes scaffolds that have been loaded with active substances with the purpose of contributing to the restoration process. After all, a section concerning the regulations applied, until now, for this kind of construct is included.

## 2. Chitosan-Based Bioinks for Tissue Engineering

Although many efforts have been made to further improve bioprinting techniques, the production of suitable bioinks for bioprinting is restricted by rheological, mechanical, and biological points of view. By preference, the rheological and mechanical characterization of bioinks should be carried out in the presence of cells as they can significantly affect the viscosity of polymer solutions and, consequently, the printability. The main point is that the printing procedure needs to be evolved at physiological conditions (pH and temperature) and also that no stress is caused on the hydrogel material itself and, thus, on the cells encapsulated in the bioink. Furthermore, cells enclosed by bioinks can reduce the shear thinning behavior during printing, often leading to phase separation and precipitation of the bioinks. For that reason, the development of new bio-ink formulations remains a challenge for researchers [14].

Due to their natural character, chitosan-based bioinks reveal high biocompatibility and low cytotoxicity. In the meanwhile, poor mechanical properties, difficulty in sterilization, and reproducibility are some restrictions of chitosan-based hydrogels used as bioinks. Modifications such as crosslinking with chemical agents or by irradiation, incorporation of thickener molecules or nanoparticles, or even functionalization of the chitosan molecule could be some solutions to enhance integrity and functionality [11]. The following research on chitosan-based bioinks that have recently been published is presented. Apart from this, Table 1 highlights the limited existing translations of these 3D CS scaffolds that limit their application to clinical care.

### 2.1. Bioinks of Composites of Chitosan Hydrogels

Current studies, in order to overcome the limitations of chitosan, combine chitosan with other materials such as PCL, D-(+) raffinose, poly(gamma glutamic acid), β-glycerophosphate, hydroxyethyl cellulose, gelatin, FRESH bath and several salts (TPP, K_2_HPO_4_, NaHCO_3_) as crosslinking molecules.

**Figure 5:**
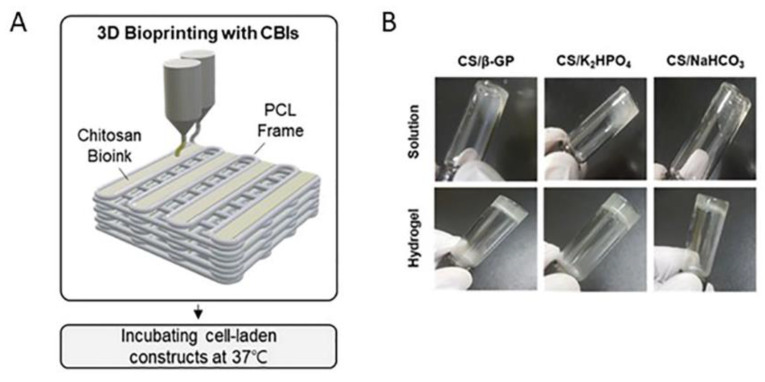
(**A**) Schematic representation of the 3D bioprinter for 3D printing and gelation using chitosan bioinks (CBIs). (**B**) Representative images of chitosan sol-gel transitions induced by temperature. Reprinted from [16].

Poly(e-caprolatone) (PCL), a biocompatible thermoplastic polymer, is often used with chitosan to enhance the mechanical properties of the final 3D structures. Li et al. investigated the capability of tetrahedral framework nucleic acid (TFNA), a DNA form that reinforces the regeneration process, in the promotion of the differentiation of synovial mesenchymal stem cells (SMSCs) and its behavior inside the network of CS hydrogel/3DP (PCL) in terms of the healing of articular cartilage (AC) damage and regeneration of cartilage defects in rabbits [15]. Firstly, CS suspension was inserted into 3DP PCL scaffolds, and then the blend formed a gel at 37 °C. Afterward, the hydrogel was enhanced, concerning the regeneration activity, with synovial mesenchymal stem cells (SMSCs). Genipin was added to the mixture for chemical crosslinking. The complex formed provided improved water absorption and appropriate pore size for cell attachment. However, more in vivo experiments should be evaluated in order to investigate the degradation rate of TFNA during time and infection issues. Altogether, this method combines the bioactive environment of chitosan material with the enhanced mechanical properties of PCL scaffolds. As previously mentioned, chitosan hydrogels printed on PCL frame were studied after using three gelling agents (β-glycerophosphate (β-GP), potassium phosphate (K_2_HPO_4_), and sodium bicarbonate (NaHCO_3_) [16]. Among the three, NaHCO_3_ showed the highest energy storage capability, and a more uneven porous morphology NaHCO_3_ agent also proves more efficient for cell attachment from day one according to cell viability assays. The use of the three gelling agents shows similar results to the human periodontal ligament stem cells (PDLSCs)-laden constructs, while for both, cell-laden or not, constructs the sol-gel transition induced at 37 °C (Figure 5). This research also noted that solvent type did not affect the gel shape and gelation time, although acetic acid seemed more biocompatible than other acids. In general, this work compares several kinds of gelling agents and solvents for the bioink of CS/NaHCO_3_ for tissue engineering.

Intini et al. prepared a 6% chitosan solution carrying D-(+) raffinose pentahydrate at 290 mM. After the printing, the scaffold cooled at −14 °C with a series of Peltier cells. Post printing, gelation occurred by incubating in a KOH (8% *w*/*v*) solution, and the construct was stored in phosphate-buffered saline (PBS) in order to keep the shape integrity of the design. Moreover, an extra sample such as the previous one but with the insertion of a full dense layer of chitosan as the bottom layer was prepared in order to facilitate the cell growth restraining the cells inside. The most optimized proliferation rate of cells was attained after 35 days on 3D scaffolds when the normal dermal human fibroblast (Nhdf) cells and aneuploid immortal keratinocyte (HaCaT) cells were combined while the elimination of the gaps in the scaffolds was observed. The research team commented that the specific scaffold provides cost benefit, owing to the starting material and also reproducible characteristics [17]. This work highlights the importance of in vivo tests for chronic dermal wounds and the cost-effectiveness of chitosan-based scaffolds as an important factor for scale-up production.

Pisani et al. reported preliminary studies on coaxial extrusion at 37 °C of chitosan (CS) and poly(gamma-glutamic acid) (Gamma-PGA). Furthermore, Fourier-transform infrared spectroscopy (FT-IR) revealed the formation of inter polyelectrolyte complex (IPEC) due to interaction between the amino groups of chitosan and carboxyl groups of gamma-PGA. Reduced time of gelation, less than 10 s, and stability for more than one month in cell culture were proved. Interestingly, bioprinted hydrogel with 6% CS was capable of maintaining the majority of the cells viable for 2 weeks. Nevertheless, further optimization by studying the effect of geometry could result in a better exchange of oxygen and nutrients inside the hydrogel [18]. This work concludes that the parameters useful to accomplish one-step 3D bioprinted IPEC by CS/Gamma-PGA were set at 37 °C temperature and 37 Pa pressure to samples with concentrations of CS 4.5% or 6% wt/vol and Gamma-PGA 2%.

**Figure 6:**
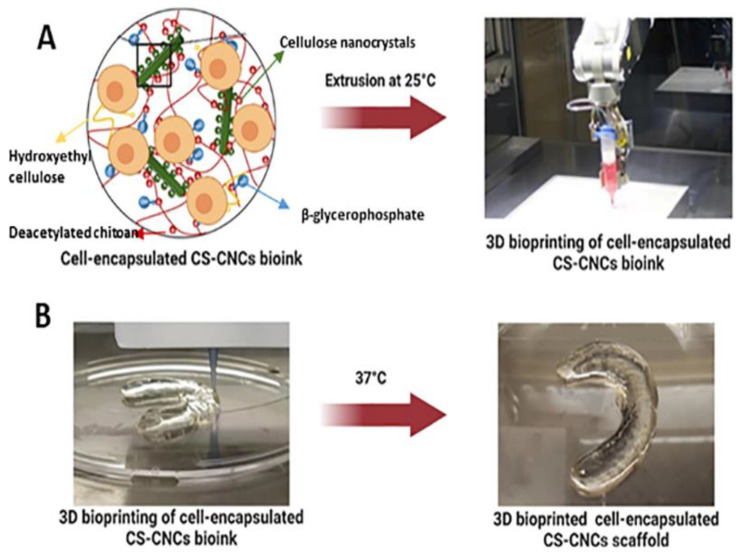
Schematic illustration of the 3D bioprinting process. (**A**) Cell-encapsulated bioink was loaded into 3D-bioprinter cartridges and bioprinted onto a cell-culture glass coverslip with cartridge temperature controlled at 25 °C. (**B**) 3D bioprinted scaffold of a patient-derived knee meniscus using the CS–CNC placebo bioink. The bioprinted scaffold was spontaneously gelled on a glass printing plate by temperature stimulation at 37 °C. Reproduced from [20]. Copyright 2021, American Chemical Society.

Maturavongsadit et al. investigated a bioink from thermogelling chitosan, glycerophosphate, hydroxyethyl cellulose, and cellulose nanocrystals (CNCs). Optimum concentrations of components were selected based on fast gelation at 37 °C (less than 7 s) (Figure 6). The research demonstrated that CNCs and pre-osteoblast cells (MC3T3-E1) ameliorated viscosity and mechanical properties. The low printing pressure (15–20 kPa) did not endanger the cell viability. Apparently, the coexistence of CNCs in the chitosan scaffolds positively affected the osteogenesis of MC3T3-E1 cells [19]. This study highlights the advantage of using CNC to thermo-/pH-responsive chitosan hydrogel for 3D bioprinted constructs for the repair of the large bone defect. Roehm et al. proposed a modification to a compact, low-priced 3D printer apparatus in order to facilitate the incorporation of the cells during printing, avoiding any toxicity from post-printing processes. Particularly, this research team explored the bioprinting of thermogelling chitosan- gelatin (CG) hydrogel with β-glycerophosphate (βGP). The importance of the preparation of the sample, including centrifugation, mixing, and degassing, was underlined as an important factor for printability and fiber formation. The shear thinning behavior was demanding for the protection of the cells during printing. Moreover, they noticed that increase in the concentration of chitosan in the mixture causes a decrease in gelation temperature, with the danger of premature gelation near 25 °C, while the increase in the time of the gelation process favors the bioprinting as it decreases the size of the fiber. It was also noted that precooling the syringe could further control temperature-dependent gelation. Cell-laden hydrogels were prepared, but further studies about their functionality must be evaluated [20]. Overall, this study presents a low-cost alternative to conventional 3D bioprinter apparatus for 3D bioprinting of thermosensitive CG hydrogels with high cell vitality.

Rahimnejad et al. introduced the use of a warm supporting bath, Pluronic based, called FRESH to help the bioprinting process of chitosan hydrogels with the use of a mix of sodium bicarbonate and β-glycerophosphate as gelling agents. Actually, the FRESH method aided the mechanical support of the structures as well as the thermal crosslinking avoiding the evaporation of the sample and facilitating its collection. Furthermore, the use of the bath improved as well, and the printing accuracy of all samples with better results at the concentration of 2% chitosan hydrogel, preventing swelling after immersion in PBS. The latter also exhibited satisfying rheological behavior and MSC survival. The FRESH method paves the way for the use of thermosensitive chitosan-based hydrogels for tissue engineering uses [21].

The research team of Hafezi attempted to optimize a previous study of their group based on a system of chitosan crosslinked with genipin. Specifically, they inserted a first layer consisted of sodium alginate as first layer with a view to make the construct more rigid. The low-pressure printing of cell-laden hydrogels enabled the viability of keratinocytes (KC) and human dermal fibroblasts (HDF) at a level greater than 88% [22].

An overview of the recent studies on chitosan-based bioinks and their application as scaffolds are also presented in Table 2.

### 2.2. Bioinks of Chitosan-Modified Hydrogels

**Figure 7:**
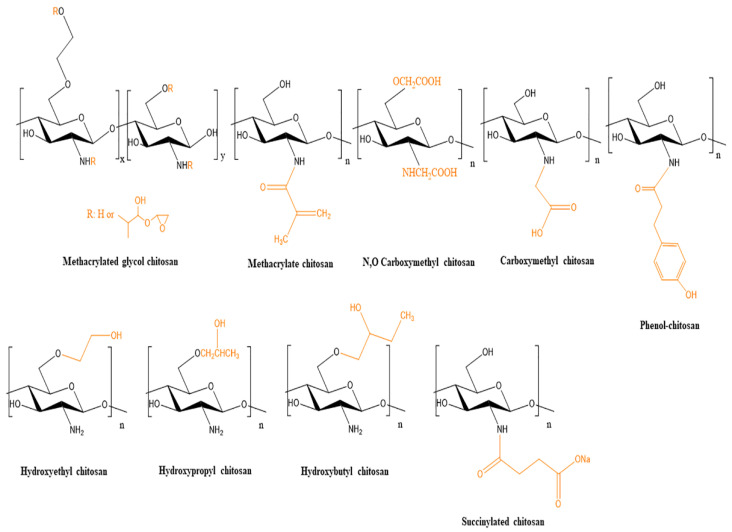
Chitosan derivatives that have been used for bioinks in 3D bioprinting.

Functionalization of the chitosan molecule could be some solution to enhance integrity and functionality. Several chitosan derivatives (methacrylated glycol chitosan, methacrylate chitosan, N,O carboxymethyl chitosan, N-Carboxylmethyl chitosan, phenol chitosan, hydroxyethyl chitosan, hydroxypropyl chitosan, hydroxybutyl chitosan, succinylated chitosan) have been recently used as bioinks and are illustrated in Figure 7.

**Figure 8:**
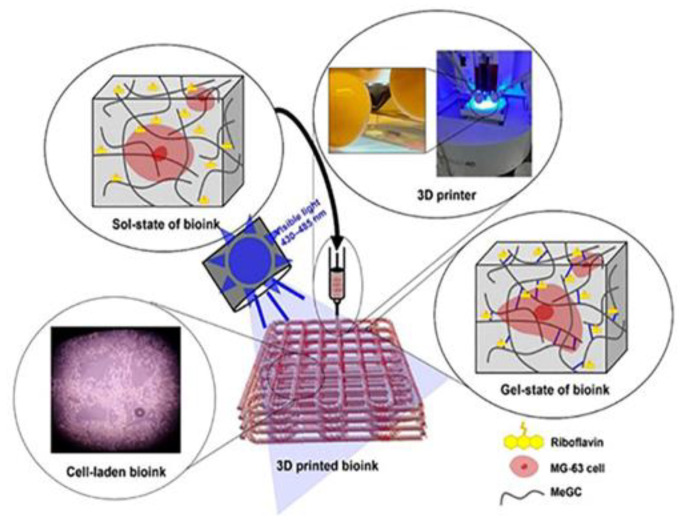
Schematic illustration for preparation for 3D bioprinting of MeGC bioink. Reprinted from [20], Copyright 2022, Elsevier.

Chang et al. trying to encounter the harmful gelation procedures of neat chitosan, prepared a bioink of the water-soluble and photo-curable methacrylate glycol chitosan (MeGC) with 1:1 ratio of amino groups of glycol chitosan (GC) to amino groups of glycidyl methacrylate (GM). Riboflavin was used as photoinitiator at visible light at 430–485 nm. The optimum shape fidelity was observed for the sample with 3% MeGC and photo-curing for 70 s (Me-GC-70) and was selected for loading with MG-63 cells (Figure 8). The results showed cell growth and remarkable bone differentiation but further in vivo tests are needed in order to make it a potentially appropriate alternative for bone tissue engineering applications [23]. The authors reported for the first time the use of visible light instead of UV irradiation for crosslinking of MeGC hydrogels, specifically MeGC-70, appropriate as bioink.

**Figure 9:**
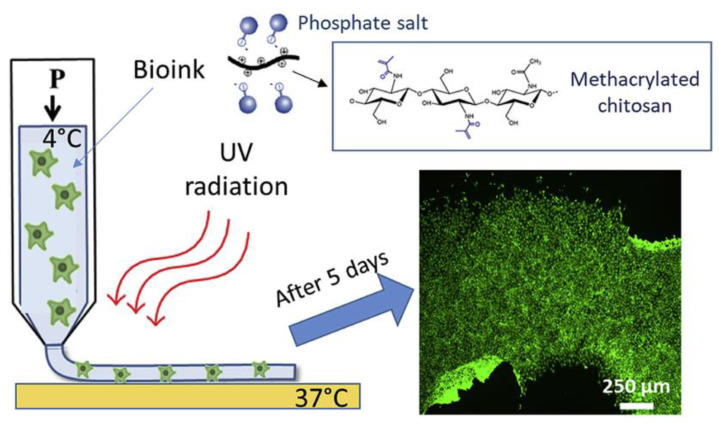
Dual-crosslinked hydrogel bioink using chitosan methacrylate together with β-glycerol phosphate with NIH 3T3 cells. Reprinted from [24], Copyright 2020, Elsevier.

Methacrylated chitosan was also explored by Tonda-Turo et al. with the further introduction of β-glycerophosphate salt (β-GP) to add thermosensitive properties. The ink showed no cytotoxicity during in vitro tests with fibroblasts (NIH/3T3), osteoblast-like cells (Saos-2), and neuronal-like cells (SH-SY5Y). Furthermore, NIH/3T3-laden scaffolds were composed and gelled under increased temperature (37 °C) and radiation of 365 nm (Figure 9). Proper biocompatible 3D structures were fabricated, highlighting it as a prominent combination for the reconstruction of complex tissues [24]. Results indicated that dual crosslinking lead to stable 3D bioprinted constructs without affecting the cell viability. Another attempt with this chitosan derivative has been made by Gaihre et al., who used methacrylated chitosan with gelatin as the medium for deposition of MC3T3-E1 pre-osteoblasts in phosphorylated-oligo [poly(ethylene glycol) fumarate] included acrylated montmorillonite (Ac-MMT) and also to facilitate the cells-Ac-MMT interaction. Results of the cell-laden constructs regarding the live/dead cell assay showed viable cells and cell growth and differentiation for 3 days. Concluding this research shed light on the combination of 3D printing and bioprinting to develop artificial cell-laden tissues for bone tissue engineering [25].

**Figure 10:**
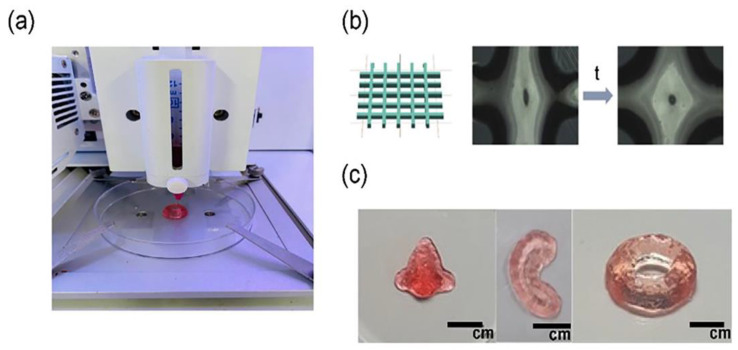
(**a**) Three-dimensional printing of self-healing OHA/GC/ADH/ALG hydrogel encapsulating ATDC5 cells. (**b**) Microscopic images of 3D-printed filaments of OHA/GC/ADH/ALG self-healing hydrogel and (**c**) 3D-printed constructs of various shapes. Reprinted from [27], Copyright 2021, Biomedicines.

The water solubility and flexibility of GC have attracted the interest of many researchers to use it as a drug carrier or imaging agent in an effort to treat cancers and microbial infections [26]. Roh et al. studied GC with OHA with the introduction of adipic acid dihydrazide (ADH), whose existence caused competition between the imine bond of OHA/GC and the acylhydrazone bond of OHA/ADH inducing chemical crosslinking. Extra crosslinking was achieved with the addition of alginate and calcium ions with the purpose of enhancing the mechanical properties. From printability studies, it was determined that printed fibers deposited were finally stacked to form a single structure owing to its self-healing ability (Figure 10). Apart from that, it was demonstrated that OHA/GC/ADH/ALG hydrogels could provide a microenvironment suitable for the chondrogenic differentiation of ATDC5 cells in vitro. This self-healing bioink system may have great potential in many biomedical applications, including tissue and organ regeneration, using a 3D printer [27]. Crosslinking with alginate and calcium ions was proved that offer further mechanical stability to this self-healing bioink system.

Carboxymethyl chitosan is an attractive chitosan derivative due to its solubility at pH range 7–9, which is favorable for cell encapsulation. Specifically, N, O Carboxymethyl chitosan (NOCC), another chitosan derivative, was used by Butler et al. as bioink due to its immediate degradation profile. Several blends of NOCC and agarose were fabricated and subsequently loaded with neuro 2A cells compared to pure NOCC cell loaded or pure agarose cell loaded. It was observed that the increase in the concentration of NOCC led to proper rheology properties and, thus, good printability. On the contrary, the increase in the content of agarose reduced the rheological properties but increased cell viability. The bioprinting at 37 °C seemed not to favor printability as the cell viability. Moreover, increased concentration of NOCC leads to less than 24 h degradation, satisfying printability but zero cell viability, while the reduced concentration of NOCC caused the opposite. Nevertheless, a balance between the two properties was achieved for the samples of 40% agarose and 60% NOCC (AG40NC60), having a prospective in tissue engineering applications [28]. Generally, the results pointed out that NOCC does not benefit the printability as the cell vitality, but an optimum combination of the two properties appeared in the AC40NC60 sample. The same chitosan derivative was also examined by another research attempted to emphasize the significance of the addition of polyphosphate (polyP) to the bioink of a NOCC-based scaffold as a forceful activator for proliferation, differentiation and mineralization of MSC in 3D printed tissue parts. The bioink was formed also with the contribution of alginate as the hydrogel part of the scaffold, which forms with ions Ca^2+^, a crosslinked hydrophilic polymer, and also gelatin served as the matrix to facilitate cell attachment and the moving of the cells. Apart from the essential metabolic energy that polyP offers for intracellular and extracellular activities, alongside, it also induces bone formation. Overall, they note that polyP enriched bioink lead to spheroidal aggregates cell forms coming out of the printed tissue parts confirming their novel composition suitable for printing implants [29]. This research attempt to emphasize the beneficial use of polyP in the bioink in the effort to resemble the properties of the extracellular matrix.

Trying to achieve printing at room temperature, structure maintenance, and reduce the time of preparation of the bioink, Chen and his team proposed an alternative way of preparation called time-sharing structure supporting (TSHSP) preparation. The system selected was aldehyde hyaluronic acid (AHA)/N-carboxymethyl chitosan (CMC) with fast gelation, so ready to use, and gelatin (GEL)/4-arm poly(ethylene glycol) succinimidyl glutarate (PEG-SG) with slow gelation, important for stability after printing. Crosslinking assisted the formation of proper integrated cell-laden constructs enduring for 21 days where the diffused cells (NE-4C, C2C12, and chondrocytes) were viable for an extended period. The proposed bioink seemed to be promising for tissue engineering and targeted cell therapy but also for a new generation of hydrogel preparation [30]. Generally, this work suggests that the TSHSP strategy is a more convenient solution to maintain not only the shape of the 3DP hydrogel but also the microstructure. CMC was further reported by Wang et al. in an effort to prepare a hybrid hydrogel from GelMA and CMC intended to regenerate blood vessels. GelMA/CMC hydrogel was further enhanced with BMSCs, which showed better mechanical behavior and configured an environment similar to ECM [31]. The formation of catechol-modified polymeric nanoparticles was proposed by Puertas Bartholome et al. for their antioxidant, anti-inflammatory, and neovascularization properties and also the capability to entrap hydrophobic drugs such as coumarin-6, which is effective for wound healing applications. The NPs were afterward homogeneously dispersed into a hydrogel of CMC and hyaluronic acid (HA), where fibroblasts were incorporated. Hence, the complex system prevails in the ability to load hydrophobic molecules, the local controlled release of the loaded nanoparticles, and the custom-made geometry formulation. Cell studies supported cell proliferation of the fibroblasts over 14 days [32]. After all, the research team of Bartholome points out the important role of catechol-modified NPs in controlling local drug administration and increasing the bioactivity of the system of the bioink. In another research, CMC was combined with amorphous calcium phosphate (ACP), labeled as CMC-ACP, leading to a composite of nanoparticles with hydrogel for bone regeneration. The novel composite display excellent biocompatibility, MSC proliferation, and cell attachment and promote osteoinductivity. In vivo tests proposed the bioprintability and the further utilization of the composite for osteoprogenitor-cell-based bone tissue regeneration [33]. This work showed, for the first time, the stabilization of ACP nanoparticles with osteoinductive properties in a CMC hydrogel in order to prepare a cell-friendly environment.

**Figure 11:**
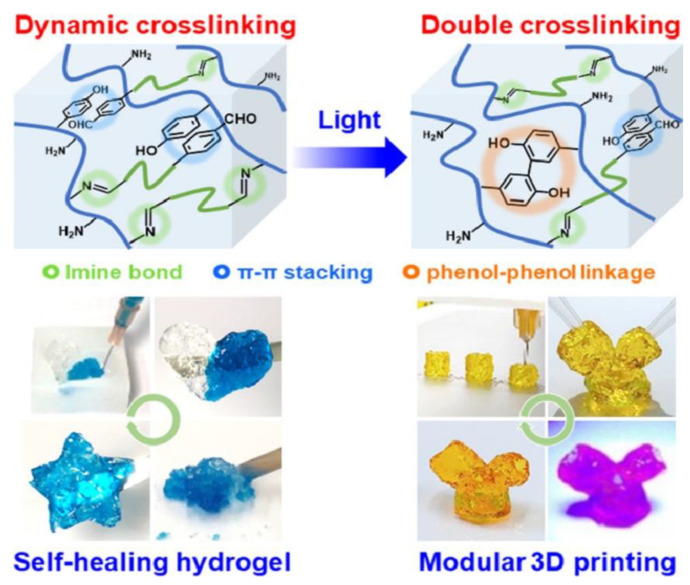
Schematic illustration for preparation of CPDP hydrogel by dynamic benzoic imine crosslinking between Chi-P and DF-PEG and after light crosslinking. Filament formation after each method of crosslinking. Reprinted from [34], Copyright 2021, Elsevier.

Liu et al. developed a self-healing hydrogel by phenol modification of chitosan (CS-Ph, which was crosslinked with dibenzaldehyde terminated telechelic poly (ethylene glycol) (DF-PEG), labeled as CPDP. This modification of chitosan enables fast and durable gelation as well as light-visible crosslinking ability. During the preparation of the hydrogel, imine bonds are formed between the amine groups of both components (Figure 11). The sequent visible light crosslinking can additionally support the hydrogel due to the forming of phenol-phenol binding. As a result, this hydrogel is well utilized for the 3D printing process. Moreover, independently printed parts can be combined into an entire construct because of the adherence and self-healing character of the hydrogel. Human mesenchymal stem cells (hMSCs)-laden hydrogels were printed and resulted in some dead cells after 4 and 24 h of culture. The researchers propose that changes in the parameters of printing and reduction of the force applied to the material could avoid the phenomenon [34]. The phenol functionalization of chitosan seems to change the physicochemical properties of CPDP hydrogel and could extend its biomedical applications.

**Figure 12:**
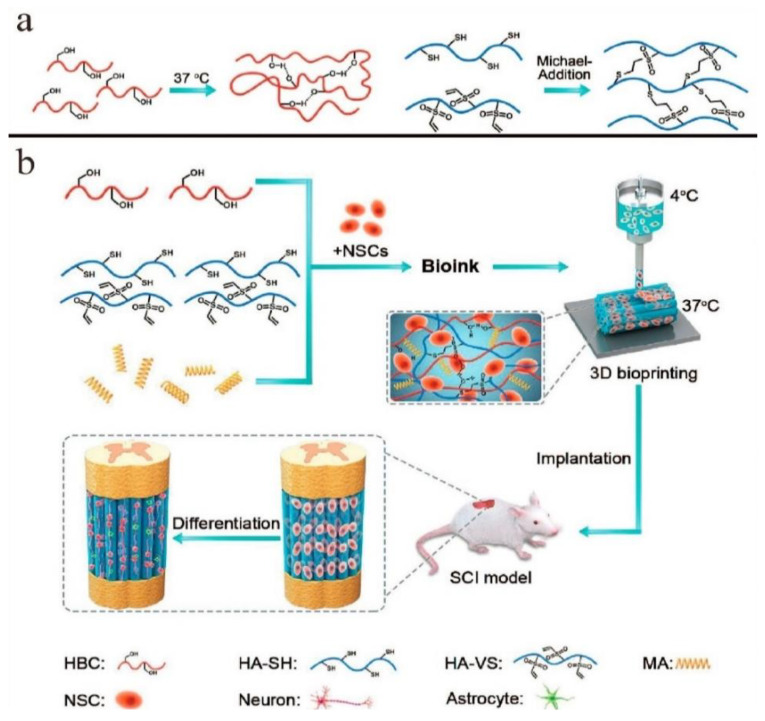
Schematic representation of the NSC-laden bioprinted neural tissue constructs for in vivo SCI repair. (**a**) The crosslinking reactions during and after the 3D printing of the HBC/HA/MA bioink and (**b**) 3D bioprinting of the NSC-laden white matter of spinal cord-like scaffold and its application for in vivo SCI repair. Reprinted from [36], Copyright 2021, Elsevier.

Nie et al. tried to overcome the limitations of thermo-responsive hydrogels concerning the cell aggregates and the rigidity, which results in limited cell viability developing a new thermosensitive hydrogel consisting of poly(N-isopropyl acrylamide) (pNiPAM) and hydroxyethyl-chitosan (HECS) loaded with dithiol-modified graphene oxide nanosheets (t-GO) (pNHG) [35]. The regulation of low critical solution temperature (LCST) could perform with varying the weight ratio of pNiPAM/HECS/t-GO while the incorporation of the human bone mesenchymal stem cells (hBMSCs) could be enacted at 20 °C. The highlight of this research is the enhancement of cell viability combined with the insert of t-GO nanosheets, which gives a potential for its use in 3D bioprinting. Neural stem cells (NSC)-laden constructs were fabricated for the first time by Liu et al. for in vivo spinal cord injury (SCI) repair in rats overcoming the difficulties of this operation. The polymeric matrix used for the incorporation of the cells was hydroxypropyl chitosan (HPC), with thiolated hyaluronic acid (HA-SG), vinyl sulfonated hyaluronic acid (HA-VS), and matrigel (MA). The whole system demonstrated a fast gel phase (20 s) at 37 °C and inherent crosslinking capacity leading to direct bioprinting of spinal cord-like constructs (Figure 11). The viability of the cells in the matrix remained extremely high, approximately 95%, and alongside the interaction of cells, polymers and neuronal differentiation were achieved [36]. This study present for the first-time in vivo results of NSC-laden bioink that also stimulates the parallel linear structure of white matter of the spinal cord. Wang et al. also used HPC in the form of microspheres together with poly(γ- glutamic acid) (PG) embedded in GelMA solution with the aim to create a multi-network hydrogel suitable as a bioink [37]. The multi-network hydrogel retained shear thinning behavior, presenting favorable extrudability and injectability even in water, remaining stable. Apart from that, this hydrogel could achieve good printability in several shapes and mechanical integrity after further UV crosslinking. On the report of SEM images, dense distribution of the microspheres in the hydrogel was observed, while the formation of them in presence of GelMA and after UV radiation was not as clear as in presence of GelMA (Figure 12). Adipose-derived stem cells (ASCs)- loaded bioinks revealed satisfying cell vitality after printing and UV application. Another water-soluble CS derivative, hyroxybutyl chitosan (HBC), was explored as a bioprinted hydrogel in combination with oxidized chondroitin sulfate (OCS). The specific hydrogel was intended for the fabrication of a cartilage repair implant, trying to overcome the harmful environment formed around the MSC-based implants and the absence of their in vivo stability. HBC/OCS hydrogels exhibited pleasant biocompatibility and an appropriate environment for human adipose-derived MSCs (HAMSCs) encapsulation and proliferation. Various inner structures and external shapes were formed with the aid of sacrificial molds. However, further optimizations to control the shape are required [38].

Another chitosan derivative, succinylated chitosan (C), was explored by Turner and his collaborators in the interest of core-shell (c/s) networks for tissue-engineered constructs (TECs) intended for wound care management. The bioink noticed consisted of gelatin methacryloyl (GelMA) as shell and peptide-functionalized, succinylated chitosan (C)/dextran aldehyde (D) cell-laden as the core. The constructs were loaded with bone mesenchymal stem cells (BMSCs) in the shell bioink and human umbilical vein endothelial cells (HUVECs) in the core bioink. After printing, microdesigns were created, presenting high cell viability followed by vessel formation. The prepared constructs favorably encapsulated and delivered mesenchymal and endothelial cells preserving an appropriate environment for cell growth, the possibility for differentiation, and the creation of tube-like structures [39]. Overall, this research promoted the development of a single-step process for the fabrication of bioink used for wound healing management.

An overview of the recent studies on chitosan-derivatives-based bioinks and their application as scaffolds are presented in Table 3.

## 3. Chitosan-Based Biomaterial-Inks

Even if the definition of the bioinks refers to an integrated 3D organized cell population, the process is complicated enough, and many factors should be taken into account. For example, cells enclosed by bioinks can reduce the shear thinning behavior during printing, often leading to phase separation and precipitation of the bioinks [14]. For that reason, the development of new bio-ink formulations remains a challenge for researchers. According to the classical tissue engineering approach of biofabrication, cells are seeded into preformulated scaffolds, optionally in cooperation with the delivery of bioactive factors that favors the ECM formation. This process includes in vitro maturation and afterward implantation to the host tissue intended for regeneration. Alternatively, in vitro 3D tissue models are developed for application in an in vitro system or screening system. A current approach includes introducing in situ stem cells into the scaffold to trigger an immune response [40]. Research on chitosan-based inks used in tissue engineering applications with potential delivery of active substances to the target tissue is presented.

### 3.1. Chitosan-Based Biomaterial Inks for Tissue Engineering Applications

#### 3.1.1. Biomaterial Inks of Chitosan and Chitosan Composites

**Figure 13:**
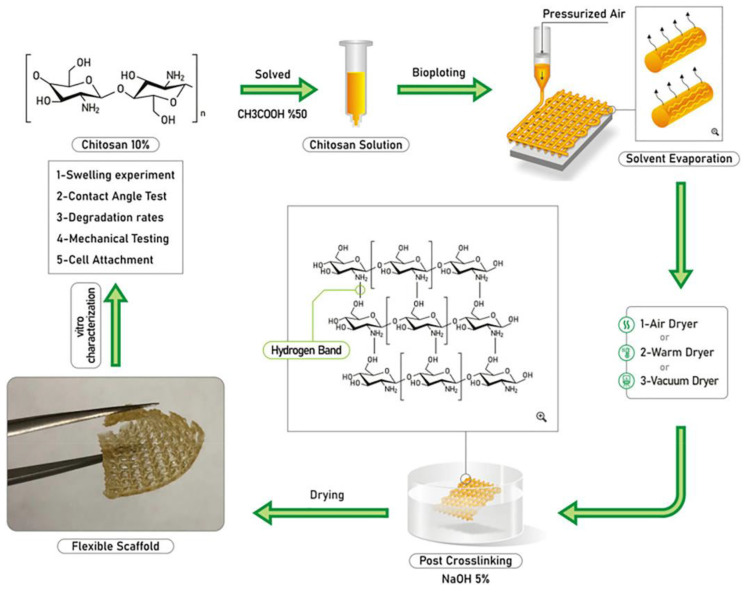
Schematic illustration representing stages including solution preparation, fabrication of 3D printed chitosan scaffolds (10% (*w*/*v*), chemical reaction, solvent evaporation, post crosslinking, and final drying. Reprinted from [41], Copyright 2020, Elsevier.

Based on several concentrations of chitosan solutions (8%, 10%, and 12%), Sadeghianmaryan et al. selected 10% as optimum for the printing of ten-layer cubic scaffolds. Characteristics of geometry were tested in respect of layer thickness and pore size, indicating smaller pore sizes than the original construction. Three types of drying methods were tested, at room temperature, 60 °C, and under vacuum, with the purpose of studying their effect on the mechanical properties (Figure 13). Scaffolds crosslinked with air-drying and those that have a 2 mm pore size appear to have the highest elastic modulus. It seems that also degradation rate and swelling properties are affected by the technique of drying, as samples with fewer concentrations and larger pores showed enhanced degradation and water retention rate. Despite the encouraging results of the cell studies, more in vitro studies must be evaluated [41]. This study shows that the drying method affects the mechanical and biological properties of the material, with the air-drying technique being suggested.

Bergonzi and his research team used CS with D-(+) raffinose, such as the previous study by Intini et al. [16], with the supplementary introduction of α-tocopherol (VitE, at concentrations of 0.1% and 1%) proposed as hydrogel dressings for chronic wounds [42]. According to the experimental results, the addition of VitE provided extra antioxidant activity and biocompatibility over 28 days of human fibroblast cultures in any of the tested scaffolds but limited antimicrobial activity on Pseudomonas aeruginosa and Staphylococcus aureus. Furthermore, the drying step assured the maintenance of the structure as far as 80% of the process with the capacity to absorb body fluids. The proposed hydrogels could be efficient as dressings for chronic wounds [42].

Gelatin exhibits the ability to form physically crosslinked hydrogels at low temperatures and cause prominent cell viability. Fischetti et al. chose mixtures of chitosan with gelatin in ratios of 1:2 and 1:3 *w*/*w*. Initially, the pH of the chitosan solution was adjusted to 4.7 to form a polyelectrolyte with the negative groups of gelatin, and after that, the pH of the mixture was adjusted to 6.5 in order to form an insoluble polymer. Tripolyphosphate (TPP) was chosen as a crosslinker, alternatively to glutaraldehyde and genipin, which are cytotoxic and costly, respectively. Because of the thermo-sensitive behavior of the mixtures, the crosslinking was applied at two temperatures, 4 °C and 37 °C. According to the study, no significant difference is observed after crosslinking procedure. Scaffolds with crosslinking at 4 °C had the most shape fidelity to the CAD dimensions due to the gel-state of the gelatin at this temperature. On the contrary, at 37 °C, the physical network of gelatin is destroyed because of the structure of the molecule. However, the scaffolds of 60 min crosslinking at 4 °C do not have the expected diameter of the fiber related to the nozzle diameter, which means the collapse of the fibers after printing something at crosslinking at 37 °C. Cytocompatibility was checked using L929 cells [43]. Overall, despite previous results, the concentration of TPP and duration of crosslinking are higher for a lower proportion of gelatin in the mixture. Wu et al. studied the hydrogel of chitosan with gelatin as a coating material on titanium alloy substrate where the concentration of chitosan varies, 3% 5% 7%, and 9%, and the concentration of gelatin is stable at 10%. Characterization techniques showed a regular macro-mesh structure and a honeycomb micro-network structure. In addition, it is indicated that the increase in the content of the mixture in chitosan results in better water absorption and level of degradation. According to the mechanical test, the optimum sample was the one with 7% chitosan and 10% gelatin. Furthermore, the adhesion test demonstrated bonding between the hydrogel coating and the under layer of titanium alloy to the sample with the highest content of chitosan (7%). It seemed that the progressive existence of chitosan in the samples also promoted the antimicrobial activity against E. coli and S. aureus after the content with the nano-silver solution. Finally, the sample with 7% chitosan appears to enhance the viability of NIH-3 T3 cells. As a result, this hydrogel can be a promising fastening material in artificial joint prostheses [44].

Silk-protein biomaterials have been used for biomedical applications due to the significant properties of silk, such as mechanical properties, biodegradability, and processability. In particular, silk in particles or fiber forms proved promising as reinforcing fillers in developing composite scaffolds due to its flexibility and its possibility to enhance tissue regeneration. Zhang et al. attempted to do a comparative relation between three types of silk reinforcing fillers, silk powder (SP), silk microfibres (MF), silk nanofibres (NF) as additives for 3D printing of chitosan hydrogels and their influence on the printability and physicochemical features of them. Biological studies further confirmed that different silk fillers, especially silk nanofibres, provided alternative methods for 3D printing of tougher hydrogel scaffolds without compromising cell adherence and proliferation capability compared to neat CS hydrogels. After all, the NF additive proved to be the best option, presented the most enhanced mechanical properties, and also promoted the best fibroblast growth appearing perspectives for the future as bioink [45].

Wu et al. assessed a composite of chitosan as the matrix and bioglass (BG) from a home-made cryogenic printing system. The addition of the BG did not influence the interconnection of the pure structure as well as the flexibility and swelling capacity. However, over 20% of BG resulted in a reduction in the mechanical properties of the scaffold even though the antibacterial (tested against S. aureus and P. aeruginosa) and cell growth performance were improved. Finally, in vivo wound healing studies of the CS/BG scaffolds in rats revealed better performance in the 3DP samples with 30% of BG than in freeze-dried ones due to structural differences. These results support that CS/BG scaffolds are a favorable option for wound healing applications suggesting a way to use avoiding cytotoxicity issues [46]. Mora-Boza et al. worked on dual crosslinked 3D scaffolds using a low concentrated ink from GelMA/CS. Firstly they applied UV curing while printing, and after post-printing, they used a novel non-toxic, ionic crosslinker, glycerylphytate (G_1_Phy), to achieve stability of the 3DP scaffold durably. In particular, the use of this specific ionic crosslinker enables cell attachment and growth. Preliminary in vitro results of the 3DP crosslinked scaffolds using L929 fibroblasts demonstrated promising biological features [47]. The use of glycerylphytate for crosslinking technique afford robust networks and prevent the requirement for additional washing steps.

#### 3.1.2. Biomaterial Inks of Polyelectrolyte Complexes of Chitosan

Chitosan hydrogels can as well form polyelectrolyte complexes (PECs) with other molecules (e.g., pectin, hyaluronic acid, alginate, etc.) The formation of PECs is based on the electrostatic interaction between oppositely charged molecules, particularly among the carboxyl groups of forms and the amino groups of chitosan. Therefore, PECs can be formed only at a pH among the pKa values of the two ionized polymers of interest. The following works highlight chitosan-based PECs applications in 3D bioprinting.

**Figure 14:**
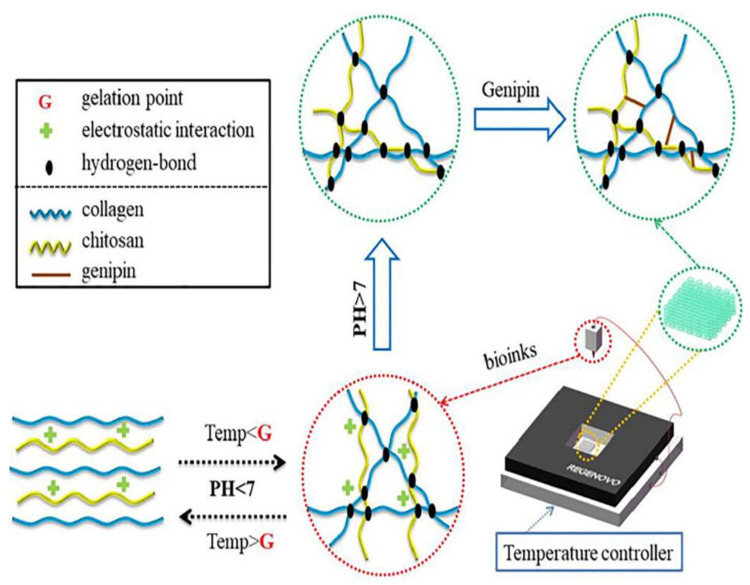
Schematic diagram of 3D printing Col/Chi scaffold. Reprinted from [48], Copyright 2021, Elsevier.

Collagen (Col) is the main structural protein in the extracellular matrix (ECM) of almost every human tissue and is composed mainly of glycine, proline, and hydroxyproline amino acids. After the rheology study, Suo et al. proved shear thinning behaviour, temperature-dependent viscosity, and gel formation from 7 to 10 °C (Figure 14). The presence of chitosan on the mixtures eliminates the water uptake of the scaffolds but ameliorates the mechanical properties, while the presence of collagen augments the degradation efficiency. Concerning the cell studies, cells were found to move to the basement of the scaffold with obvious viability. The sample with the same concentration of chitosan and collagen is concentrated in the center of the filaments, perhaps because of a more solid and smoother surface. To further observe the behavior of the cells, the scaffolds were cut vertically, where it is proved that the increase in the chitosan leads to more dead cells as a result of higher mechanical strength, as it is also shown that softer hydrogels are more friendly for cells. In general, Col/CS bioinks have a predictable porosity, which facilitates the development of cells and also the transfer of their nourishments [48]. In this study, despite the fact that in most of the cases, the swelling ratio of the hydrogels is increasing during the decrease in the degradation rate, Col/CS hydrogels appear to have similar swelling, correlated to structure maintenance, while their degradation rate is different. The highlight of the study is the concentration of the cells to the bottom of the construct, maybe due to a denser structure and flattened surface. The same combination of materials with a ratio of Col/CS 0.36/1.00 were used by Heidenreich et al. to study their rheology and printability. The crosslinking was performed with EDC/NHS before printing in order to obtain stable scaffolds, while the gelation after printing was achieved through the addition of NaHCO_3_ followed by 37 °C incubation of the 3D printed scaffolds. Even though the degradation tests of the scaffolds performed in PBS with collagenase constructs were stable after 44 h while non-toxicity was proved in NIH-3T3 fibroblasts, further studies are needed for multi-layered scaffolds [49]. It was indicated that in situ nebulization immediately after the printing or pre-crosslinking to low concentration inks might be a solution for multi-layered scaffolds of Col/Chi hydrogel. Apart from that, the control of the viscosity by keeping the mixture in ice depends on the time.

**Figure 15:**
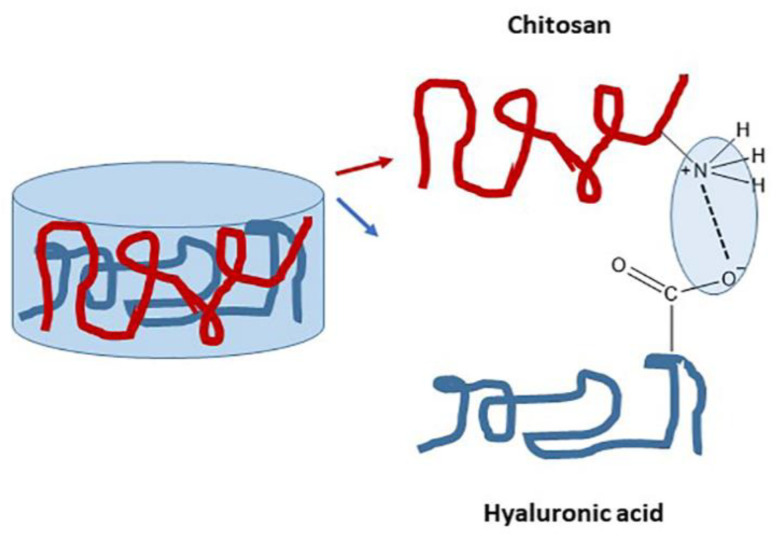
Electrostatic interactions between chitosan and hyaluronic acid [51].

Hyaluronic acid is another polysaccharide that can be blended with chitosan and form a polyelectrolyte complex (Figure 15). Vieira de Suza et al. prepared a thermosensitive hydrogel consisting of different concentrations of chitosan and hyaluronic acid for cartilage tissue engineering. The non-toxicity of the hydrogels was proved, but there was a difficulty with the attachment of the cells on the hydrogel substrate, and also their migration, maybe due to hydrogel density and charges coexistence. Preliminary cell studies suggested a more ‘cell-friendly’ gel for the cells when hyaluronic acid coexists. Further studies are expected in order to confirm this hydrogel as a possible bioink, especially concerning how the preparation procedure influences the attached cells [50]. Promising results went out from this research concerning the cytotoxicity of CS/HA hydrogels, but interactions between cells and polymers or the density of the hydrogel seem to put difficulties on the circulation of the cells.

**Figure 16:**
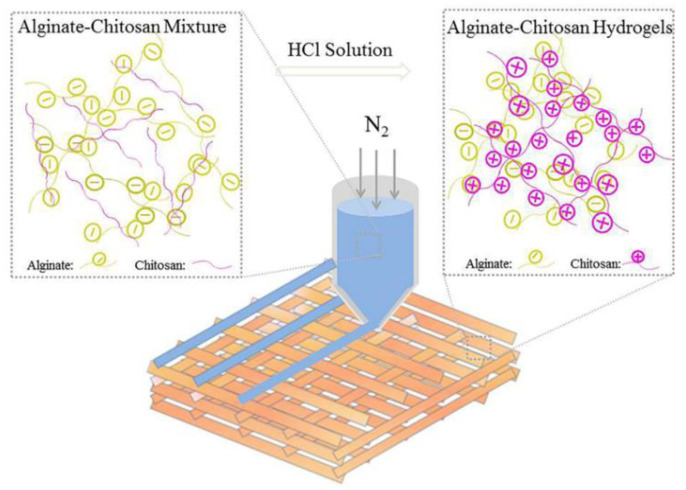
Schematic diagram of three-dimensional (3D) printing alginate–chitosan polyion complex hydrogels. Reproduced from [52].

Alginate is a biocompatible anionic polymer derived from brown algae and composed of units of β-d-mannuronic acid (M blocks) and its epimer α-l-guluronic acid (G blocks). It presents high biocompatibility and shear thinning behavior that is commonly used in 3D bioprinting. However, alginate solutions have low viscosity, which is an obstacle to the maintenance of shape fidelity of alginate-based 3D printed constructs. Liu et al. proposed the preparation of polyelectrolyte complexes from chitosan and alginate-based on interactions between amino groups and carboxyl groups of alginate, respectively. The blending of the system took place on neutral pH chitosan, which constitutes a viscosity modifier. Post printing, the construct was sprayed with HCl solution in order to achieve better shape fidelity because of the conversion of the amino groups to ammonium and, as a result, more intense interaction with alginate (Figure 16). The hydrogel produced afforded an appropriate environment for cell growth of hASCs where their differentiation could enact tissue regeneration [52]. The important thing is that by regulating the pH directly after printing, the complexation between chitosan and alginate is enhanced, improving the viscosity of the blend.

#### 3.1.3. Biomaterial Inks of Modified Chitosan 

**Figure 17:**
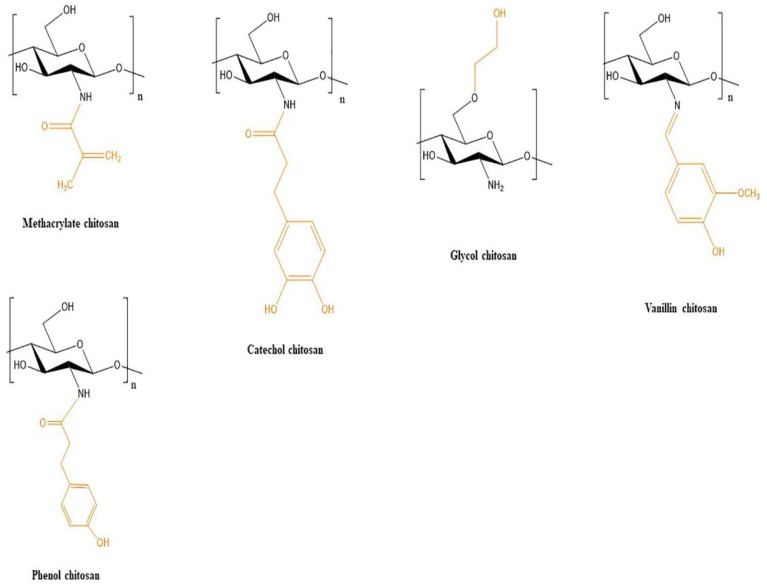
Chitosan derivatives have been used as biomaterial inks in 3D bioprinting.

Except for cell-laden constructs, some other CS derivatives have recently been used as printable materials appearing to be biocompatible with cell cultures after printing. The CS derivatives(methacrylate chitosan, catechol chitosan, glycol chitosan, and vanillin chitosan discussed in this review are illustrated in Figure 17.

**Figure 18:**
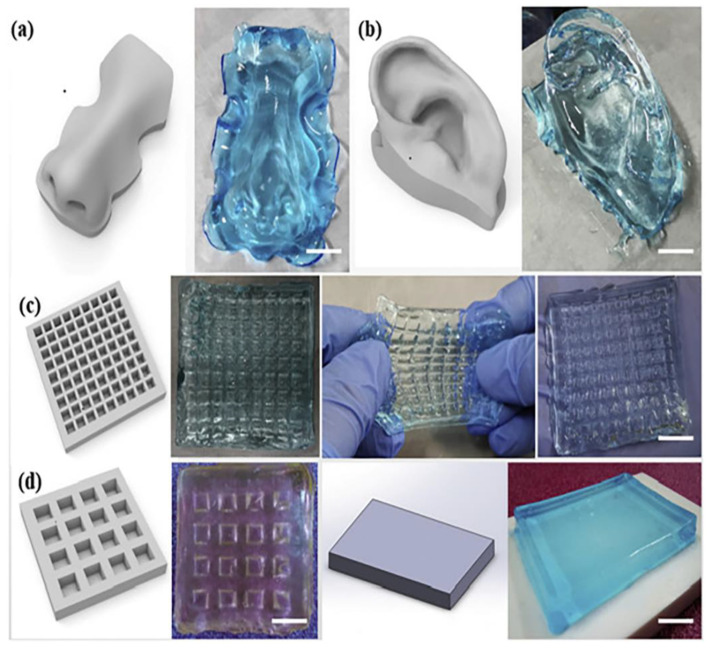
(**a**) DLP based 3D printing of CHIMA/PAM bioinks. (**b**) the CAD model and the printed construct of the nose. (**c**) the CAD model and the printed construct of the ear auricle with the helical fold. (**d**) Lattice structures of CHIMA/PAM hydrogels with different mesh size produced by DLP. Printed constructs were not damaged when they were stretched. Bar: 10 mm. Reprinted from [54], Copyright 2021, Elsevier.

The preparation of bioinks consisted of methacrylate-modified chitosan (ChMA) and methacrylate gelatin (GelMA) immersed in nanohydroxyapatite (nHap) were studied by Osi et al. Different concentrations of GelMA in the mixtures were tested with regard to control the viscosity and stability of the initial samples. Double crosslinking was applied physically with the use of temperature and chemically with photo crosslinking for further stability. Even if the incorporation of hydroxyapatite was for the enhancement of the biological activity and mechanical properties, it finally did not affect enough these properties of the final hydrogels. For the cell studies, printed hydrogels were immersed in rat-bone-marrow-derived stem cells (BMSCs) culture [53]. Mainly, in this work, it was reconfirmed that dual crosslinking results in high printing resolution. A photo curable bioink composed of methacrylated chitosan and acrylamide (AM) was provided for DLP bioprinting achieving complex structures of hydrogels and mechanically stable (Figure 18). It was confirmed that the gelation time depended on the photoinitiators while the mechanical properties and cytotoxicity were AM ratio depending. Compared to neat PAM and ChMA hydrogels, the hybrid hydrogel proposed appeared ameliorated compression strength, flexibility, and advantageous biocompatibility. Coming together, ChMA/PAM in an appropriate ratio could be beneficial as a bioink [54]. In this work, it is proven that DLP bioprinting can be a good alternative for the formation of more complicated structures with higher elastic modulus and desirable biocompatibility.

**Figure 19:**
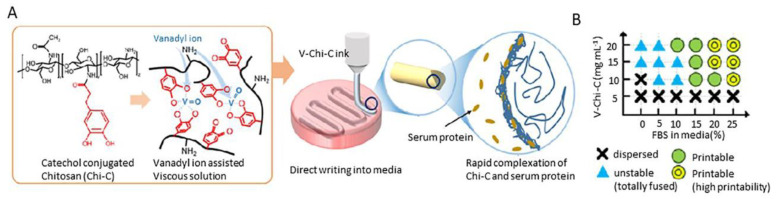
(**A**) Catechol-chitosan ink with vanadyl ions (V-Chi-C). (**B**) Preparation of the ink and 3D printing process. Reproduced from [55] Copyright 2018, The Royal Society of Chemistry.

Lee et al. developed a bioink of catechol-conjugated chitosan (Chi-C) as a surface coating for biomedical devices (Figure 19). This bioink was directly printed into the fetal bovine serum (FBS) medium. The author reported the use of direct printing in serum-containing media by immediate complexation of Chi-C and serum proteins without the appliance of any external factor. The shape fidelity succeeded through complexes formatted between the Chi-C and the proteins from the serum. In order to increase the mechanical properties of the bioink, they inserted a slight amount of vanadyl ions via metals into the system, which also favored cell viability at 90%. The scaffolds were stable for 7 days at 37 °C [55]. This work shows that the introduction of proteins in the chitosan-based formulation enacts the development of interactions leading to more stable complexes.

**Figure 20:**
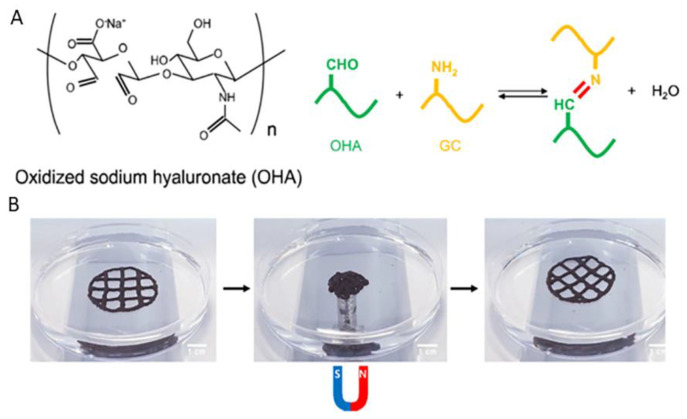
(**A**) Schiff base formation between OHA and GC. (**B**) Change in the 3D-printed structure by application of a magnetic field. The original shape was recovered after removal of the magnetic field. Reprinted from [56], Copyright 2020, Elsevier.

Oxidized polysaccharides carry aldehyde groups that can form imine bonds with the amino groups of chitosan (Schiff base reaction), followed by the formation of hydrogels. Ko et al. investigated a ferrogel with self-healing properties consisting of glycol chitosan (GC), ameliorated water solubility, and oxidized hyaluronate (OHA) containing superparamagnetic iron oxide nanoparticles (SPION). This ferrogel was used as a 3D ink but had a great perspective on 4D printing as it changes its shape in the case of the application of a magnetic field and returns to the original shape in case of its absence (Figure 20) [56]. A Schiff derivative of chitosan with vanillin (VACS) combined with neat chitosan to regulate the viscosity was studied for 3D printing applications by Michailidou et al. [57]. In this study, it was confirmed that the air-drying method affects the behavior of the scaffold. Freeze-dried samples tend to have higher swelling ability compared to room-temperature-dried scaffolds because of the increased porosity achieved via the freeze-drying process. The growth of fibroblasts on uncrosslinked samples of CS-PEC scaffolds, dried at room temperature, was enhanced compared to crosslinked ones.

For the first time, Hidaka et al. proposed an ink from Phi-chitosan photocrosslinked with sodium persulfate (SPS) and tris (2,2-bipyridyl) ruthenium (II) chloride (Ru(bpy), which could be applied as inks for both extrusion-based and vat polymerization based bioprinting. Three-dimensional printing hydrogel constructs with fast gelation (10 s) and printing accuracy were obtained. The results propose this ink as a promising material for biomedical applications. It was displayed that adjusting the proportion of SPS and Ru(bpy) in the chitosan hydrogels affected the gelation time and the mechanical properties. Antimicrobial activity against E. coli and S. aureus supported the fact that the photo-cured chitosan-Ph hydrogels appear to have antimicrobial properties even after gelation. In addition, the biodegradability of the material showed potential use in biomedicine as there is no need for removal [58]. It was demonstrated that the selection of appropriate photocrosslinkers for water-soluble Chi-P can lead to an appropriate ink for both extrusion-based and vat polymerization-based bioprinting.

### 3.2. Chitosan-Based Biomaterial Inks for Active Substance (API)-Loaded Scaffolds

In some cases, in order to integrate bioactive characteristics, 3D printed chitosan-based scaffolds include several active ingredients (drugs, proteins, growth factors, etc.) that they release in their surrounding environment during their application. Most of the recent research on chitosan-based inks concerns wound healing and bone tissue engineering applications. Concerning the first, the formulation of the inks should offer control of bleeding and, ideally, the ability to deliver antimicrobial and anti-inflammatory substances to prevent infections and pain. According to the following, such substances could be casein, fluorescein sodium, mupirocin, lidocaine, and Manuka honey. Regarding bone tissue engineering, many growth factors, such as bone morphogenic proteins (BMPs) and vascular endothelial growth factors (VEGFs), have been explored for repairing bone defects. Except for that, antibiotics such as levofloxacin could prevent infection [59].

#### 3.2.1. Biomaterial inks of Loaded Chitosan Composites

**Figure 21:**
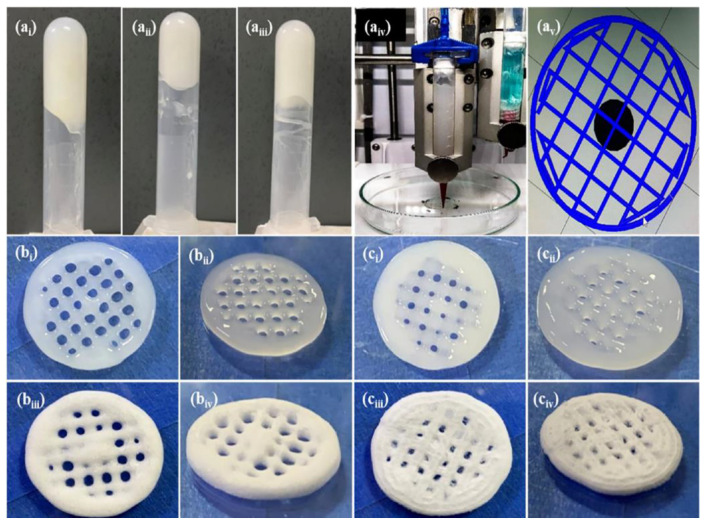
Digital images of TCNF gel 1.25 wt.% (**a_i_**), 1.75 wt.% (**a_ii_**), and TCNF/casein conjugate (1.75 wt.%) (**a_iii_**); 3D printed model and printing process (**a_iv_**,**a_v_**), 3D printed TCNF ((**b_i_**) front view), ((**b_ii_**) side view) and TCNF/casein ((**b_iii_**) front view), ((**b_iv_**) side view); freeze-dried 3D printed TCNF ((**c_i_**) front view), ((**c_ii_**) side view) and TCNF/casein after cross-linking with chitosan ((**c_iii_**) front view), ((**c_iv_**) side view). Adapted with permission from [60]. Copyright 2022, American Chemical Society.

In an attempt to control traumatic hemorrhage, Biranje et al. focused on 3D- TEMPO-mediated oxidized cellulose nanofibrils (TCNF)/Casein-based scaffold. The attachment of the casein to TCNF was carried with the aid of EDC N-(3-dimethylaminopropyl)-N’-ethyl carbodiimide hydrochloride (EDC) and N-hydroxysuccinimide (NHS), while the printed scaffold was further crosslinked with chitosan through ionic complexation (Figure 21). The coexistence of TCNF, casein, and chitosan in a 3D mixture benefits cell adhesion, the multiplying and decreasing of the clotting time. Rheology test confirmed viscoelastic behavior and shape stability of the scaffold. The TCNF/casein scaffold exhibited quickening clotting of red blood cells (RBCs) and platelet adhesion compared to neat TCNF scaffold and also commercial products from cellulose. In addition, the cytocompatibility of the samples was performed by a 3D cell culture study and showed that the composite prepared scaffold enhances the viability and the reproduction of NIH 3T3 fibroblast cells, which is crucial for wound healing. This work reveals that the explored composite 3D scaffold is a promising construct not only for its biodegradation capacity but also for the control of bleeding [60]. The citrate modification of the iron nanoparticles and then their immersion in a chitosan hydrogel was held by Lin et al. [61]. Covalent bonds that were created between citrate groups of the nanoparticles and the CS molecule contributed to the immobilization of the nanoparticles inside the hydrogel. The loading and the release of bovine serum albumin (BSA) were further explored by the authors.

Poly(ethylene glycol) (PEG) is another water-soluble synthetic polymer frequently applied in pharmaceutical and biomedical applications. Hafezi et al. created multi-layered wound dressings constituted by crosslinked chitosan with genipin, glycerol (GLY), or poly ethylene glycol (PEG) as a plasticizing agent. The optimal 3D printed hydrogel, with a 1:1 ratio of CS: PEG and 1% of genipin, concerning the ductility, appeared to have good mucoadhesive properties. Moreover, this optimum 3D printed formulation had the ability to maintain the moisture of the wound and at the same time provide the release of the model drug, fluorescein sodium (FS), even though not in a controlled way to prevent the frequent replacement of the dressing. Finally, the results of the MTT assay indicated biocompatibility with human fibroblast cells and satisfying cell viability after 48 h (>70%) [62]. The option of genipin as a crosslinker has benefits such as biocompatibility due to less toxicity and, at the same time, anti-inflammatory and antibacterial properties.

The research group of Ergul investigated scaffolds from chitosan and polyvinyl alcohol (CS/PVA) containing several different concentrations of hydroxyapatite (HAp) and loaded with bone protein (BMP-2). The scaffold with 15% HA, loaded with the growth factor or not, was proved biocompatible with human mesenchymal stem cells (MSCs) and appropriate for the printing of 36 layers scaffold, promising as a bone implant [63]. As shown, the insertion of Hap in chitosan-based hydrogels in a certain percentage seems to increase its biocompatibility. Deng et al., in their effort to design a system for the delivery of the rhBMP-2 growth factor, prepared an implanted complex of poly(lactic-co glycolic acid) (PLGA)/nanohydroxyapatite (nHAp)/CS. In more detail, rhBMP-2 was loaded on CS microspheres that were subsequently incorporated in a thermosensitive CS hydrogel derived from CS and β-glycerophosphate. PLGA/nHAp and the CS hydrogel were put in a two-barrel 3D bioprinter where one barrel had the PLGA/nHAp freeze-dried material with temperature regulated at 130 °C, the other for CS hydrogel at 4 °C. The scaffold was implanted into a rabbit mandibular defect model and showed slow release and induction of the differentiation and proliferation of osteoblasts [64]. Since the resultant scaffold of PLGA/nHap/CS is managed effectively to control the delivery of rhBMP-2, it can be suggested as a bone-forming formulation.

Chen et al. fabricated pastes compounded by (CS/Gel/HAp) for 3D printing for bone tissue engineering. After the printing process, the scaffolds were coated with chitosan and sodium hyaluronate. Protein BMP-2 and growth factor VEGF were incorporated into the composite scaffolds simply by absorption. A constant release of both substances was found to happen during the first 14 days. Cell studies demonstrated the capability of these scaffolds to provide osteogenic differentiation of MC3T3-E1 cells and, thus, bone formation [65]. Similarly, to the previous study, 3D printed CS/Gel/Hap hydrogel could deliver BMP-3 and VEGF, after their simple absorption from the matrix, in a controllable way in order to induce bone formation. Marques et al. evolved an ink formed by high-loaded biphasic calcium phosphate (ΒCP) (45%) and chitosan to print levofloxacin (LEV)-loaded scaffolds for bone regeneration. The crosslinking process was double, ionically because of calcium ions and covalently with genipin. The optimum scaffold had 80% HA and 20% β-TCP and was proposed for dual function, bone regeneration, and infection management. Levofloxacin-loaded scaffolds exhibited a premature burst release making them more appropriate for rapid local administration, while at the same time, they showed inhibition of bacterial growth [66]. After all, the initial fast release of LEV-loaded scaffolds could be beneficial for localized drug delivery and local tissue regeneration.

**Figure 22:**
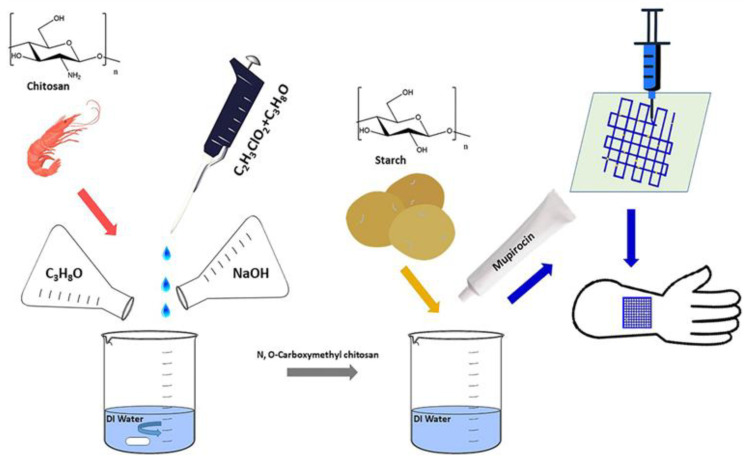
Three-dimensional printing of starch NOCC wound dressing scaffolds.

Naseri et al. combined NOCC with starch and drugs loaded with mupirocin, an antibiotic drug used for skin infections (Figure 22). The ratio of NOCC to starch seemed to manipulate the release rate of mupirocin, showing that a higher quantity of starch is accompanied by less amount of drug released but a more sustained profile compared to neat NOCC matrices. This behavior is in respect to the fact that starch is less hydrophilic than the NOCC. Finally, an antibacterial study against S. aureus demonstrated increased inhibition zones for the samples with higher content of the modified chitosan [67]. Consequently, modifications of chitosan, such as NOCC, can improve its solubility and give the opportunity for 3D printing in low temperatures and organic solvent-free, protecting anti-infective factors sensitive to heat that may be carried. He et al. attempted a modification to chitosan incorporating ethylenediaminetetraacetic acid (EDTA) in order to add a carboxyl group and achieve physical crosslinking with calcium ions and, thus, mechanical strength. A bioink blending modified and neat chitosan was created and loaded with chondrocyte cells. Cell studies confirmed favorable cell viability while rheology and mechanical properties proved dependent on the constituents’ ratio, and adjustments could expand the use of this bioink in tissue engineering [68]. Through the modification of the chitosan, enhanced mechanical properties were obtained, while physical crosslinking constitutes an alternative process for low toxicity.

#### 3.2.2. Biomaterial Inks of Loaded Chitosan Derivatives or Polyelectrolyte Complexes of Chitosan

**Figure 23:**
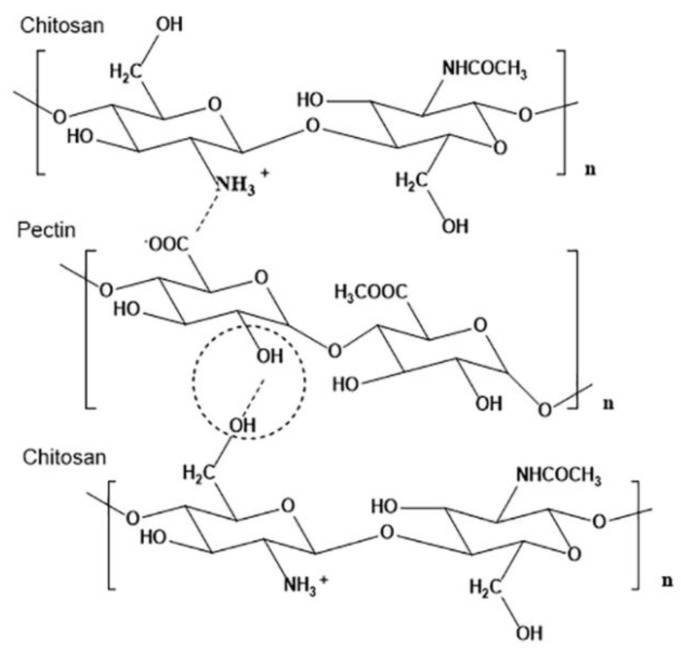
Physical interactions between chitosan and pectin.

Pectin, a broadly used ingredient in the pharmaceutical and food industries, can also be printed. Liu et al. prepared 3D scaffolds from chitosan and chitosan crosslinked with pectin and genipin, printed on NaOH solution substrate, and then loaded with pentoxifyllin, a model anti-inflammatory drug [69]. Fully interconnected and well-controlled pores were obtained by 3D printing compared to traditional freeze-dried scaffolds. Cell studies concluded that the scaffolds from chitosan with pectin (CP) were the most biocompatible samples from the total studied with osteoblast cells. Apart from that, they presented the most enhanced alkaline phosphatase (ALP) activity which triggered the mineralization process and finally the apoptosis by day 21 of the test. It was accepted that 3D plotted samples are stiffer than freeze-dried ones and subsequently less degradable and suitable as bone scaffolds. Chitosan-pectin (CS-PEC) scaffolds were fabricated by Long et al. for wound dressing application for lidocaine delivery. As reported by FTIR spectroscopy, there was no electrostatic interaction between CS and PEC due to the absence of charge in PEC in the existing pH, but the development of hydrogen bonds during gelation (Figure 23). This thermosensitive gelation procedure resulted in the destruction of the hydrogen bonds during heating (at 37 °C) and reformation during cooling. The release of the lidocaine was held abruptly for the first 60 min and followed by a sustained release up to approximately 6 h as a consequence of the sponge-like network. This type of release favors the instant relief of the pain of a recent wound [70]. This kind of release is interesting when the challenge is to provide an efficient way to alleviate the pain of a fresh wound. The research group of Andriotis attempted to increase the antimicrobial activity of pectin gels with Manuka honey by incorporating inclusion complexes of chitosan and cyclodextrin with propolis extract (CCP) in order to create dressings for the treatment of ulcers and wounds. Compared to neat film from pectin, the addition of the CCP, in a certain proportion, to the samples led to a correlating increase in the time of degradation and the bioadhesiveness. However, in higher percentages of CCP, the cell viability was reduced to less than 10% *w*/*w* [71]. The acceleration of healing in burned tissues by propolis, also referred to in the literature [72], was further confirmed. Overall, this study managed to effectively show the prospective of biodegradable, 3D printable inks for the preparation of direct and indirect wound dressings.

Promising results went out from this research concerning the cytotoxicity of CS/HA hydrogels, but interactions between cells and polymers or the density of the hydrogel seem to put difficulties on the circulation of the cells. Except for the above, Sheila Maiz-Fernandez and her team have reported polyelectrolyte complexes (PECs) of chitosan-based hydrogels combined with hyaluronic acid with self-healing properties. The study concentrated on the optimization of the CHI/HA samples regarding the concentration of the components and the complexation time, where 2% and 24 h were selected, respectively. The system was loaded with sodium diclofenac and rifampicin, with the latter appearing to have a lower release rate because of its low water solubility and the bigger size of the molecule. Being biocompatible, this system could be used as a 3D fabricated scaffold and personalized injectable implant [51]. According to this work, the complexation time of PECs of CS/HA proved to affect properties such as mucoadhesiveness, rheology, and mechanical stability. The self-healing property of this hydrogel contributes to the durability of its lifetime, reducing the cost of replacement.

In general, carbonaceous materials are often utilized in biomedical applications, among others, thanks to their large surface area and the ability to overcome biological barriers [73]. Cheng et al. investigated the BMP delivery from BMP-loaded graphene oxide nanoparticles, which were then placed in a CS/Col hydrogel (10/1 *w*/*w* ratio) [74]. A drug release study showed successful release from the printed structure, in appropriate concentrations, for about 1 month with a gradual rate.

## 4. Regulatory Aspects

The technology of bioprinting is still in a very early stage but rapidly developing with a wide range of research from printing engineering to tissue engineering and cell sciences. However, before the widespread use of 3D technologies in clinical practices, some regulations should be addressed in terms of safety and quality [75]. In December 2017, the FDA announced a guidance document entitled “Technical Considerations for Additive Manufactured Medical Devices”, which give initial regulatory insight into the requirements of 3D pharmaceutical materials and biomedical devices. Consequently, many medical devices found their way into the market, but the only pharmaceutical product approved by the regulatory authorities was Spritam, a 3D printed tablet by Aprecia Pharmaceuticals, approved by the Food and Drug Administration (FDA) for the treatment of epilepsy. However, there is uncertainty about whether the directions adjust to all processes and parameters or only to the final product. In addition, according to the European Commission (EC) and European Medicines Agency, gene therapies, somatic cell therapies, and tissue-engineered products are labeled as advanced therapy medicinal products (ATMPs). Regulations of the EC for the particles of ATMPs might be applied to different phases of the 3D bioprinting process and concern assessment of product quality, efficacy, and safety [76].

Given the existing circumstances, opportunities are created at the early stage of drug development, as current legislation can be more flexible regarding 3D printing incorporation. For instance, many contract research organizations have authorized manufacturing licenses and quality release protocols and have conditions for preparation on site. In that manner, the product under testing could be manufactured in-house or in an external manufacturing facility and then delivered to the clinical test site for spreading. However, 3D printers still have some restrictions regarding the passage from laboratory to scale-up production, which could affect the translation of the formulation to later stages of development (clinical phases II and III) [76]. For example, the existing regulatory frameworks do not adequately consider the view that computer-aided 3D bioprinting can be counted as an industrial manufacturing process with the scope to fabricate personalized tissue-engineered combination products using an automated way rather than an extemporaneous, facilitating the validation. As known, the existing regulation in the EU and the USA emphasizes that combining synthetic scaffolds with cultured human cells creates a combination medicinal product that will command a high level of a regulatory investigation, particularly for the manufacturer that makes both integral parts [77]. In the EU, the regulation appears more restricted than in the USA and requires the device component to receive a separate license (CE mark) in addition to the review of the market authorization application (MAA) for the cell-based component.

Concerning the drug delivery devices, commonly, the printed DDD must be made in accordance with the current chemistry, manufacturing, and control (CMC) standards as published in FDAs 21 CFR 200 and 300 series and other relevant guidance; in the same way, the conventional medical devices/products require. In personalized drug therapy, the complication of drug delivery devices (DDD) and dosage forms lead to concerns such as liability, intellectual rights, and data protection that must be addressed for the protection of manufacturers and patients [78].

Clinically, the implementation process is assumed to be much more complex and time-consuming. One of the main regulatory discussions in this category is also based on the fact that 3D printing must be considered an extemporaneous preparation or a manufacturing process. Extemporaneous preparation means the preparation of the drug by a pharmacist, in community and hospital pharmacies, in case the requested drug is not promptly available. This easily describes the concept of the production of personalized medicines at the point of care. The co-contribution of pharmacies with the pharmaceutical industry seems to be an effective way to bring 3D printing appears to be the best path to transfer this technology into practice. Moreover, adjustments to the commercially available 3D printers should occur adaptations in commercially available 3D printers in order to be in accordance with the safety and quality demand for drug preparation. Even if there are limitations referring to the speed of 3D printers compared to traditional industrial machines, 3D printers are here to fill the therapeutic gap relating to the request for personalized therapies. In that regard, this technology can be used as a supplementary or alternative way to the production of conventional therapies. Nevertheless, in limited production, at compounding pharmacies, the difference in speed between manual preparation and the use of a 3D printer is negligible. Overcoming the limitation of speed, multiple printing heads could be used at the same time until the point that the resolution is not influenced [79]. Other challenges refer to the selection of technology cells and appropriate material used during the process [80].

New guidelines are supposed to make an appearance in the next period of time in order to set up pre-determined targets and continuous flow to the process that combine both the needs of the patient and the manufacturers. In addition, researchers should also play a key role in researching new applications with 3D technology in terms of hospital and pharmaceutical fields. In general, for incorporating 3D technology into pharmaceutical technology, an interdisciplinary project with the cooperation of regulators, scientists, manufacturers, and engineers should be constructed [81]. In total, bioprinting could play a significant role in the pharmaceutical and medicinal field, reducing the high cost and long lead times that the development process takes. Therefore, it deserves special attention and a legal framework.

## 5. Expert Opinion and Future Perspectives

Bioinks are an essential component of bioprinting and typically consist of biomaterials, mainly hydrogels, cells, or cell aggregates or their combinations. Several natural polymers, such as chitosan, have been used as bioinks. Despite the fact that several attempts have been performed for the progress of bioprinting technology, the development of new materials for bioinks combining the mechanical, rheological, and biological requirements has been restricted until now. Specifically, for chitosan, efforts are being made to the development of new gelation techniques to improve not only the chitosan handling and functionality but, at the same time, the cell-encapsulation ability. Additionally, more work is needed concerning the models and standards of bioinks materials for comparison of the developed systems. Furthermore, important is the standardization of the bioinks during the printing process to conform to their use after. With regard to the cells, the sources used for the extraction of them for the bioprinting also need to be defined for purity and functionality reasons.

The next step for this technology is in situ printing [82], where the construction of the functional biological system will be developed directly to the injured site via a machine or device, a process particularly useful for skin regeneration or bone repair. These in situ printing techniques try to print biological systems in an easy and accurate way; however, the clinical studies of in vivo bioprinting are yet to be determined. Furthermore, another evaluation of the technology is the possibility of studying the 3D printed construct during 4D printing. The shape, functions, features, and mechanisms involved in 3DBP are also evaluated in 4DBP with different stimuli for the development of controlled or sustained delivery systems. Conformational changes are observed in the structures when different stimuli are applied. Four-dimensional printing may contribute and extend the applications of in situ 3DBP resulting biological structures and scaffolds in vivo [83].

## 6. Conclusions

3D printing and bioprinting is a promising strategy for the artificial synthesis of biological structures or therapeutic delivery systems in reduced time compared to conventional methods. Specifically, the application of chitosan hydrogels in 3D printed pharmaceutical formulations provides important advantages related to biocompatibility and biodegradability of the prepared formulations. Concerning their application as implants and scaffolds for tissue engineering, these benefits are indispensable. Chitosan-based systems have a wide variety of properties that makes it possible to use them in a large number of 3D printing technologies. Nevertheless, this field is an emerging technology, and the research study in the bibliography is still limited. Furthermore, the use of natural products is restricted to the role of the excipient. Moreover, several modifications based on the functional groups of chitosan and its combination with other substances (formation of composites, complexes, etc.) could ameliorate its features. The incorporation of active substances as drugs or cell cultures into the bioinks predicts a very promising future for these products in 3D printed systems.

## Figures and Tables

**Table 1:** Translations of 3D CS scaffold presented in this review.

Sample	Animal Model	Days of Testing	Application
CS-PCL	Rabbits (*n* = 8 knees for each category)	Test of mechanical properties 3 and 6 months after surgery	Cartilage regeneration of articular cartilage
CS-D-(+) raffinose	Adult female Wistar rats	After 7, 10 and 14 days the animals were sacrificed	Wound healing evaluation in experimental diabetes in rats
CMC-amorphous calcium phosphate	Athymic nude mice (*n* = 5)	After 4, 6 and 8 weeks after implantation the animals were sacrificed	Bone regeneration
PNiPAM/HECS/GO	Sprague–Dawley (SD) rats	After 1, 7, 14 and 28 days the animals were sacrificed	General drug delivery and tissue engineering applications
HBC/HA/MA	Sprague-Dawley (SD) rats (*n* = 8)	Test of movement ability after week 12 post surgery	Spinal cord injury repair

**Table 2:** Summary of the recent studies on chitosan-based bioinks.

	Other Compounds	Cell Type	Bioink Formulation	Bioprinting Method	Condition	Application	Ref.
CS	PCL	Synovial mesenchymal stem cells (SMSCs) (3 × 10^7^ cells/mL)	CS: 2% *w*/*v*), β-glycerophosphate: 56% *w*/*v* Crosslinker: genipin: 0.5% *w*/*v*	Extrusion based	Nozzle: 26 G Temperature: 37 °C	Cartilage regeneration	[15]
CS	PCL	Human periodontal stem cells (PDLSCs) (5 × 10^5^ cells/mL)	β-glycerophosphate: 56% (*w*/*v*), Crosslinker: sodium bicarbonate: 3.5%	Extrusion based	Temperature: framework printing: 65 °C, cell-laden CS solution: 37 °C	-	[17]
CS	D-(+)raffinose	Human fibroblasts (Nhdf) and Keratinocytes (HaCaT) (1 × 10^5^ cells/mL)	CS: 6% *w*/*v*, D-(+) raffinose pentahydrate: (290 mM) Crosslinker: KOH (8% *w*/*v*)	Extrusion based	Nozzle: 26 G Temperature: 25 °C	Diabetic wound healing	[16]
CS	Poly(gamma-glutamic acid)	Human adult fibroblasts (2 × 10^5^ cells/mL)	CS: 4% or 6% *w*/*v* PGA: 2% *w*/*v*	Extrusion based	Nozzle: 22 G for chitosan and 25 G for Gamma-PGA Pressure: CS: 25–40 kPa Gamma-PGA: 5–10 kPa Temperature: 37 °C Speed: 600 mm/min	-	[18]
CS	Cellulose nanocrystals, Hydroxyethyl cellulose	Pre-osteoblast cells (MC3T3-E1) (5 × 10^6^ cells/mL)	CS: 3% *w*/*v* CNCs: 0–2% *w*/*v* HEC: 0–0.5 mg/mL Crosslinker: β-GP: 100 mM	Extrusion based	Nozzle: 20 G Pressure: 20 kPa Temperature: room temperature Speed: 2 mm/s	Bone tissue engineering	[19]
CS	Gelatin, 2GP	IMR-32 cells from neuroblastoma (CCL-127) (2 × 10^6^ cells/mL)	Chitosan: 2% *w*/*v* Gelatin: 4% *w*/*v* 2GP: 0.56% *w*/*v* Crosslinker: genipin	Extrusion based	Temperature: 37 °C	-	[20]
CS	Gelatin	Human bone marrow mesenchymal stems cells (MSCs) (5 × 10^6^ cells/mL)	Chitosan: 2% or 3% *w*/*v* Gelatin: 2% *w*/*v* Crosslinker: β-glycerophosphate/sodium bicarbonate: 0.1 M/0.075 M	Extrusion based	Nozzle: 25 G Temperature: printhead: room temperature, printbed: 37 °C Speed: 3–11 mm/s	-	[21]
CS	Gelatin, PEG	Human dermal fibroblasts(HDF) and Keratinocytes (KC) (0.5 × 10^6^ cells/mL)	Chitosan: 0.5% *w*/*v* Gelatin: 1% *w*/*v* PEG: 2% *w*/*v* Crosslinker: genipin 1% *w*/*v*	Extrusion based	Nozzle: 22 G Presure: 25 kPa Speed: 4 mm/s	Skin regeneration	[22]

**Table 3:** Summary of the recent studies on chitosan derivatives-based bioinks.

CS Derivative	Other Compounds	Cell Type	Bioink Formulation	Bioprinting Method	Condition	Application	Ref.
MeGC	-	Human osteosarcoma cell line (1 × 10^6^ cells/mL)	MeGC: 3% *w*/*v*, riboflavin: 12 μΜ Crosslinker: UV for 70 s	Extrusion based	Nozzle: 26 G Pressure: 120 ± 10 kPa Temperature: 25 °C Speed: 6 mm/s	Bone tissue engineering	[23]
CS-MA	β-glycerophosphate, lithium phenyl-2,4,6-trimethylbenzoylphospinate	Fibroblasts (NIH/3T3) (10^6^ cells/mL), osteoblast-like cells (Saos-2) and neuronal-like cells (SH-SY5Y)	CS-MA: 1.5 % *w*/*v*, βGP:0.5 g/mL, LAP: 0.05% *w*/*v* Crosslinker: UV	Extrusion based	Nozzle: 25 G Temperature: Printhead: 4 °C Printing bed: 37 °C Speed: 10 mm/s	-	[24]
CS-MA	Phosphorylated-oligo [poly(ethylene glycol)fumarate], Acrylated montmorillonite, Methyl cellulose, Gelatin	Pre-osteoblast cells (MC3T3-E1) (1.5 × 10^6^ cells/mL)	MEGC: 2% *w*/*v* MMT: 0%, 1%, 4%, 6% Irgacure 2959 1.5 wt.% of total polymer content Crosslinker: UV	Extrusion based	Nozzle: 25 G Pressure: 50 kPa Temperature: 25 °C printing bed: 4 °C Speed:10 mm/s	Bone tissue engineering	[25]
GC	Oxidized hyaluronate, Adipic acid dihydrazide, Alginate	ATDC5 cells (1 × 10^7^ cells/mL)	GC: 1 wt.% OHA: 2 wt.% ADH: 0.3% ALG 0.3 wt.% Crosslinker: CaCl_2_ 60 mM	Extrusion based	Nozzle: 25 G Pressure: 300 N Temperature: 25 °C	-	[27]
NOCC	Agarose	Neuro2a mouse Neuroblastoma Cells (1 × 10^5^ cells/mL)	Agarose: 40% NOCC: 60%	Extrusion based	Nozzle: 0.41 mm Temperature: 37 °C Speed: 3 mm/s	-	[28]
NOCC	Gelatin, Na-Polyphosphate NPs, Alginate	Mesenchymal stem cells (MSC) (3 × 10^5^ cells/mL)	NOCC: 50 mg/mL Gelatin: 25 mg/mL Na-polyP: 100 μg/mL Alginate: 1% Crosslinker: CaCl_2_ 2.5% *w*/*v*	Extrusion based	Temperature: 21 °C Pressure: 1 bar Temperature: room temperature Speed: 18 mm/s	-	[29]
CMC	Aldehyde hyaluronic acid, Gelatin, 4-arm poly(ethylene glycol) succinimidyl glutarate	NIH/3T3 fibroblasts CRL1658 and C2C12 myoblasts	AHA: 1% *w*/*v* CMC: 0.75% *w*/*v* GEL: 1% *w*/*v* PEG-SG: 0.5% *w*/*v*	Extrusion based	Nozzle: 25 G Temperature: 10 °C Speed: linear: 6 mm/s extrusion: 0.07 mL/min	-	[30]
CMC	Methacryloyl gelatin	Bone marrow mesenchymal stem cells (BMSCs) (3 × 10^5^ cells/mL)	CMC: 4% *w*/*v* LAP: 0.3% *w*/*v* 10% GelMA/0.5% CMCS 10% GelMA/1% CMCS 10% GelMA/2% CMCS	Extrusion based	Nozzle: 160 μm Pressure: non cellular inks: 0.8–1.2 bar cell-laden: 0.5–1 bar Speed: Cell-laden: 10–20 mm/s Cell-free: 15 mm/s Temperature: 16 °C	Vascular tissue engineering	[31]
CMC	Oxidized and non-oxidized hyaluronic acid	Fibroblasts (L929) (1 × 10^6^ cell/mL)	CMC: 2 wt.% HA: 0.4 wt.% Oxidized HA: 4 wt.% Catechol NPs: 2 and 29 M % Crosslinker: FeCl_3_ 20 mM	Extrusion based	Nozzle: 200 μm Speed: plunger: 0.06 mm/s, printhead: 15 mm/s	-	[32]
CS-Ph	Dibenzaldehyde- terminated telechelic poly(ethylene glycol)	Human mesenchymal stem cells (hMSCs) (4 × 10^6^ cells/mL)	Chi-Ph: 1% Crosslinker:DF-PEG 1%, UV (440–460 nm)	Extrusion based	Nozzle: 26 G	Bone tissue engineering	[34]
HBC	Thiolated hyaluronic acid, Vinyl sulfonated hyaluronic acid, Matrigel	Neural stem cells (NSCs)	HBC: 3% *w*/*v*, HA-SH: 0.3% *w*/*v*, HA-VS: 0.3% *w*/*v*, MA: 0.1% *w*/*v*	Extrusion based	Nozzle: 260 μm Temperature: printhead: 37 °C printbed: 10 °C, Pressure: 20 kPa	Neural tissue engineering	[36]
HPC microspheres	Methacryloyl gelatin, poly(γ-glutamic acid)	Adiposed derived stem cells (ASCs)	GelMA: 5 wt.%, HPC-microspheres:50 mg/mL, PG-microspheres: 50 mg/mL, I2959 Crosslinker: UV	Extrusion based	Nozzle: 18 G Temperature: 25 °C Speed: 5 mm/s	-	[37]
HBC	Oxidized chondroitin sulfate	Bone-marrow mesenchymal stem cells (BMSCs) (5 × 10^6^ cells/mL)	GelMA: 13% *w*/*v*, Irgacure 2959: 0.2% *w*/*v*, Peptide/CD gel concentration: 0.02%/8% SC/0.6% DA, Crosslinker: UV	Extrusion based	Nozzle: core: 400 μm, shell: 800 μm, temperature: printhead: 25 °C, printbed:16 °C, Pressure: core: 225 kPa, shell: 150 kPa, Speed: 5 mm/s	Wound healing	[38]

## Data Availability

Not applicable.

## Abbreviations

AC	Articular cartilage
AcMMT	Acrylated montmorillonite
ACP	Amorphous calcium phosphate
ADH	Adipic acid dihydrazide
AG	Agarose
AHA	Aldehyde hyaluronic acid
ALP	Alkaline phosphatase
AM	Acrylamide
ATMPs	Advanced therapy medicinal products
BCP	Biphasic calcium phosphate
BG	Bioglass
BMP	Bone morphogenic protein
BMSCs	Bone mesenchymal stem cells
C	Succinylated chitosan
CAD	Computer aided design
CCP	Chitosan cyclodextrin with propolis extract
CG	Chitosan crosslinked with genipin
Chi-C	Catechol-conjugated chitosan
Chi-Ph	Phenol chitosan
ChMA	Chitosan methacrylate
CMC	Carboxymethyl chitosan
CNC	Cellulose nanocrystals
CP	Chitosan crosslinked with pectin
CS	Chitosan
D	Dextran aldehyde
DDD	Drug delivery devices
DDS	Drug delivery system
DLP	Digital light processing
EC	European Commission
EDC	N-(3-dimethylaminopropyl)-N’-ethyl carbodiimide hydrochloride
EDTA	Ethylenediaminetetraacetic acid
ECM	Extracellular matrix
FBS	Fetal bovine serum
FDA	Food and Drug Administration
FRESH	Freeform reversible embedding of suspended hydrogels
FS	Fluorescein sodium
FTIR	Fourier
G_1_Phy	glycerylphytate
Gamma-PGA	Poly(gamma-glutamic acid)
GC	Glycol chitosan
Gel	Gelatin
GelMA	Methacrylate gelatin
Gly	Glycerol
GM	Glycidyl methacrylate
GO	Graphene oxide
HA	Hyaluronic acid
HaCaT	Aneploid immortal keratinocyte cells
HAMSCs	Human derived mesenchymal cells
HAp	Hydroxyapatite
HA-SG	Thiolated hyaluronic acid
HBC	Hydroxybutyl chitosan
hBMSCs	Human bone mesenchymal stem cells
HDF	Human dermal fibroblasts
HECS	Hydroxyethyl chitosan
HMSCs	Human adipose-derived mesenchymal stem cells
HUVECs	Human umbilical vein endothelial cells
ION	Iron oxide nanoparticle
IPEC	Inter polyelectrolyte complex
KC	Keratinocytes
K_2_HPO_4_	Potassium phosphate
LCST	Low critical solution temperature
LEV	Levofloxacin
MA	Matrigel
MAA	Market authorization application
MeGC	Methacrylate glycol chitosan
MF	Silk microfibres
MG-63	Osteosarcoma cell line
MSCs	Mensechymal stem cells
NaHCO_3_	Sodium bicarbonate
nanoHAp	Nanohydroxyapatite
NF	Silk nanofibres
Nhdf	Normal dermal human fibroblast cells
NIH/3T3	Fibroblasts
NHS	N-hydroxysuccinimide
NOCC	N,O Carboxylmethyl chitosan
NPs	Nanoparticles
NSCs	Neural stem cells
OCS	Oxidized chondroitin sulfate
OHA	Oxidized hyaluronate
PAM	Poly(acrylamide)
PBS	Phosphate-buffered saline
PCL	Poly(e-caprolactone)
PDLSCs	Periodontal ligament stem cells
Pec	Pectin
PECs	Polyelectrolyte complexes
PEG	Polyethylene glycol
PEG-SG	Poly(ethylene glycol) succinimidyl glutarate
PgGA	Poly(gamma-glutamic acid)
PLA	Poly(lactic acid)
PLGA	Polylactic-co glycolic acid
pNiPAM	Poly(N-isopropyl acrylamide)
polyP	Polyphosphate
RBC	Red blood cells
rhBMP	Recombinant human bone morphogenic protein
Saos-2	Osteoblast-like cells
SCI	Spinal cord injury
SH-SY5Y	Neuronal-like cells
SMSCs	Synovial mesenchymal stem cells
SP	Silk powder
SPECs	Surfactants and polyelectrolytes complexes
SPS	Sodium persulfate
TCNF	TEMPO-mediated oxidized cellulose nanofibrils
TECs	Tissue engineering constructs
TEMPO	2,2,6,6-tetramethylpiperidinyl-1-oxyl
TFNA	Tetrahedral framework nucleic acid
TPP	Tripolyphosphate
VACS	Vanillin chitosan
VitE	α-tocopherol
βGP	β-glycerophosphate
